# The management of intra-abdominal infections from a global perspective: 2017 WSES guidelines for management of intra-abdominal infections

**DOI:** 10.1186/s13017-017-0141-6

**Published:** 2017-07-10

**Authors:** Massimo Sartelli, Alain Chichom-Mefire, Francesco M. Labricciosa, Timothy Hardcastle, Fikri M. Abu-Zidan, Abdulrashid K. Adesunkanmi, Luca Ansaloni, Miklosh Bala, Zsolt J. Balogh, Marcelo A. Beltrán, Offir Ben-Ishay, Walter L. Biffl, Arianna Birindelli, Miguel A. Cainzos, Gianbattista Catalini, Marco Ceresoli, Asri Che Jusoh, Osvaldo Chiara, Federico Coccolini, Raul Coimbra, Francesco Cortese, Zaza Demetrashvili, Salomone Di Saverio, Jose J. Diaz, Valery N. Egiev, Paula Ferrada, Gustavo P. Fraga, Wagih M. Ghnnam, Jae Gil Lee, Carlos A. Gomes, Andreas Hecker, Torsten Herzog, Jae Il Kim, Kenji Inaba, Arda Isik, Aleksandar Karamarkovic, Jeffry Kashuk, Vladimir Khokha, Andrew W. Kirkpatrick, Yoram Kluger, Kaoru Koike, Victor Y. Kong, Ari Leppaniemi, Gustavo M. Machain, Ronald V. Maier, Sanjay Marwah, Michael E. McFarlane, Giulia Montori, Ernest E. Moore, Ionut Negoi, Iyiade Olaoye, Abdelkarim H. Omari, Carlos A. Ordonez, Bruno M. Pereira, Gerson A. Pereira Júnior, Guntars Pupelis, Tarcisio Reis, Boris Sakakhushev, Norio Sato, Helmut A. Segovia Lohse, Vishal G. Shelat, Kjetil Søreide, Waldemar Uhl, Jan Ulrych, Harry Van Goor, George C. Velmahos, Kuo-Ching Yuan, Imtiaz Wani, Dieter G. Weber, Sanoop K. Zachariah, Fausto Catena

**Affiliations:** 1 Department of Surgery, Macerata Hospital, Macerata, Italy; 2 Department of Surgery and Obstetrics/Gynaecology, Regional Hospital, Limbe, Cameroon; 3 Department of Biomedical Sciences and Public Health, Unit of Hygiene, Preventive Medicine and Public Health, Università Politecnica delle Marche, Ancona, Italy; 4 Trauma Service, Inkosi Albert Luthuli Central Hospital and Department of Surgery, Nelson R Mandela School of Clinical Medicine, Durban, South Africa; 5 Department of Surgery, College of Medicine and Health Sciences, UAE University, Al-Ain, United Arab Emirates; 6 Department of Surgery, College of Health Sciences, Obafemi Awolowo University, Ile-Ife, Nigeria; 7 General Surgery Department, Papa Giovanni XXIII Hospital, Bergamo, Italy; 8 Trauma and Acute Care Surgery Unit, Hadassah Hebrew University Medical Center, Jerusalem, Israel; 9 Department of Traumatology, John Hunter Hospital and University of Newcastle, Newcastle, New South Wales Australia; 10 Department of General Surgery, Hospital San Juan de Dios de La Serena, La Serena, Chile; 11 Department of General Surgery, Rambam Health Care Campus, Haifa, Israel; 12 Acute Care Surgery at The Queen’s Medical Center, John A. Burns School of Medicine, University of Hawai‘i, Honolulu, USA; 13 Department of Surgery, Maggiore Hospital, Bologna, Italy; 14 Department of Surgery, Hospital Clínico Universitario, Santiago de Compostela, Spain; 15 Department of General Surgery, Kuala Krai Hospital, Kuala Krai, Kelantan Malaysia; 16 Emergency Department, Niguarda Ca’ Granda Hospital, Milan, Italy; 17 Department of Surgery, UC San Diego Medical Center, San Diego, USA; 18 Emergency Surgery Unit, San Filippo Neri’s Hospital, Rome, Italy; 19 Department of Surgery, Tbilisi State Medical University, Kipshidze Central University Hospital, T’bilisi, Georgia; 20 Shock Trauma Center, University of Maryland School of Medicine, Baltimore, USA; 21 Department of Surgery, Pirogov Russian National Research Medical University, Moscow, Russian Federation; 22 Department of Surgery, Virginia Commonwealth University, Richmond, VA USA; 23 Division of Trauma Surgery, Department of Surgery, School of Medical Sciences, University of Campinas (Unicamp), Campinas, SP Brazil; 24 Department of General Surgery, Mansoura Faculty of Medicine, Mansoura University, Mansoura, Egypt; 25 Department of Surgery, Yonsei University College of Medicine, Seoul, Republic of Korea; 26 Department of Surgery, Hospital Universitário Terezinha de Jesus, Faculdade de Ciências Médicas e da Saúde de Juiz de Fora, Juiz de Fora, Brazil; 27 Department of General and Thoracic Surgery, University Hospital Giessen, Giessen, Germany; 28 Department of Surgery, St. Josef Hospital, Ruhr University Bochum, Bochum, Germany; 29 Department of Surgery, Ilsan Paik Hospital, Inje University College of Medicine, Goyang, Republic of Korea; 30 Division of Acute Care Surgery and Surgical Critical Care, Department of Surgery, Los Angeles County and University of Southern California Medical Center, University of Southern California, Los Angeles, CA USA; 31 Department of General Surgery, Faculty of Medicine, Erzincan University, Erzincan, Turkey; 32 Clinic for Emergency Surgery, Medical Faculty University of Belgrade, Belgrade, Serbia; 33 Department of Surgery, Assia Medical Group, Tel Aviv University Sackler School of Medicine, Tel Aviv, Israel; 34 Department of Emergency Surgery, Mozyr City Hospital, Mozyr, Belarus; 35 Departments of Surgery, Critical Care Medicine, and the Regional Trauma Service, Foothills Medical Centre, Calgary, Alberta Canada; 36 Department of General Surgery, Division of Surgery, Rambam Health Care Campus, Haifa, Israel; 37 Department of Primary Care and Emergency Medicine, Kyoto University Graduate School of Medicine, Kyoto, Japan; 38 Department of Surgery, Edendale Hospital, Pietermaritzburg, Republic of South Africa; 39 Abdominal Center, University Hospital Meilahti, Helsinki, Finland; 40 II Cátedra de Clínica Quirúrgica, Hospital de Clínicas, Facultad de Ciencias Medicas, Universidad Nacional de Asuncion, Asuncion, Paraguay; 41 Department of Surgery, University of Washington, Seattle, WA USA; 42 Department of Surgery, Pt BDS Post Graduate Institute of Medical Sciences, Rohtak, India; 43 Department of Surgery, Radiology, University Hospital of the West Indies, Kingston, Jamaica; 44 Department of Surgery, University of Colorado, Denver Health Medical Center, Denver, CO USA; 45 Department of Surgery, Emergency Hospital of Bucharest, Bucharest, Romania; 46 Department of Surgery, University of Ilorin, Teaching Hospital, Ilorin, Nigeria; 47 Department of Surgery, King Abdullah University Hospital, Irbid, Jordan; 48 Department of Surgery and Critical Care, Universidad del Valle, Fundación Valle del Lili, Cali, Colombia; 49 Division of Emergency and Trauma Surgery, Ribeirão Preto Medical School, Ribeirão Preto, Brazil; 50 Department of General and Emergency Surgery, Riga East University Hospital ‘Gailezers’, Riga, Latvia; 51 Emergency Post-operative Department, Otavio de Freitas Hospital and Hosvaldo Cruz Hospital, Recife, Brazil; 52 General Surgery Department, Medical University, University Hospital St George, Plovdiv, Bulgaria; 53 Department of Aeromedical Services for Emergency and Trauma Care, Ehime University Graduate School of Medicine, Ehime, Japan; 54 Department of General Surgery, Tan Tock Seng Hospital, Tan Tock Seng, Singapore; 55 Department of Gastrointestinal Surgery, Stavanger University Hospital, Stravenger, Norway; 56 First Department of Surgery - Department of Abdominal, Thoracic Surgery and Traumatology, General University Hospital, Prague, Czech Republic; 57 Department of Surgery, Radboud University Medical Center, Nijmegen, The Netherlands; 58 Trauma, Emergency Surgery, and Surgical Critical Care Harvard Medical School, Massachusetts General Hospital, Boston, USA; 59 Trauma and Emergency Surgery Department, Chang Gung Memorial Hospital, Taoyuan City, Taiwan; 60 Department of Surgery, Sheri-Kashmir Institute of Medical Sciences, Srinagar, India; 61 Department of Trauma Surgery, Royal Perth Hospital, Perth, Australia; 62 Department of Surgery, Mosc Medical College, Kolenchery, Cochin, India; 63 Department of Emergency Surgery, Maggiore Hospital, Parma, Italy; 64 Department of Clinical Medicine, University of Bergen, Bergen, Norway

**Keywords:** Intra-abdominal infections, Sepsis, Peritonitis, Antibiotics

## Abstract

Intra-abdominal infections (IAIs) are common surgical emergencies and have been reported as major contributors to non-trauma deaths in the emergency departments worldwide.

The cornerstones of effective treatment of IAIs are early recognition, adequate source control, and appropriate antimicrobial therapy. Prompt resuscitation of patients with ongoing sepsis is of utmost important.

In hospitals worldwide, non-acceptance of, or lack of access to, accessible evidence-based practices and guidelines result in overall poorer outcome of patients suffering IAIs.

The aim of this paper is to promote global standards of care in IAIs and update the 2013 WSES guidelines for management of intra-abdominal infections.

## Background

The world’s burden of emergency surgery diseases is significant and appears to be increasing. Emergency services and acute surgical care constitute a major gap in the focus of the health sector worldwide, and several issues need to be addressed in order to promote a global dialogue on what is the most appropriate way to configure acute care surgery worldwide [1]. Although variations in the spectrum of surgical diseases are observed among and within countries, “essential” surgery and anaesthesia in emergency should be viewed as a core group of services that can be delivered within the context of universal access [1–3]. Particularly for the rural populations in low- and middle-income countries (LMICs), there are enormous gaps in access to life-saving and disability-preventing surgical services [4–6]. Furthermore, many hospitals continue to have logistic barriers associated with the application of evidence-based practice. This may lead to an overall poorer adherence to international guidelines, making them impractical to a large part of the world’s population [7, 8].

Complicated intra-abdominal infections (cIAIs) are an important cause of morbidity and mortality, particularly if poorly managed. A recent multi-center observational study, conducted in 132 medical institutions worldwide during a 4-month period (October 2014–February 2015) enrolled 4553 patients with cIAIs [1]. The overall mortality in this study was 9.2% (416/4533).

The aim of these guidelines is to present an evidence-based international consensus position on the management of IAIs, from collaboration of a panel of experts, with a view to promoting the standards of care for the management of IAIs worldwide.

## Methods

These guidelines have been formulated by international collaboration and discussion among an expert panel of clinicians, practicing in the field of emergency surgery. These consensus guidelines have been facilitated and coordinated by the board of the World Society of Emergency Surgery and are an update of the 2013 WSES guidelines on this topic.

The statements are formulated and graded according to the Grading of Recommendations Assessment, Development and Evaluation (GRADE) hierarchy of evidence from Guyatt and colleagues [9], summarized in Table 1.

**Table 1:** Grading of Recommendations Assessment, Development and Evaluation (GRADE) hierarchy of evidence from Guyatt et al. [9]

Grade of recommendation	Clarity of risk/benefit	Quality of supporting evidence	Implications
1A			
Strong recommendation, high-quality evidence	Benefits clearly outweigh risk and burdens, or vice versa	RCTs without important limitations or overwhelming evidence from observational studies	Strong recommendation, applies to most patients in most circumstances without reservation
1B			
Strong recommendation, moderate-quality evidence	Benefits clearly outweigh risk and burdens, or vice versa	RCTs with important limitations (inconsistent results, methodological flaws, indirect analyses, or imprecise conclusions) or exceptionally strong evidence from observational studies	Strong recommendation, applies to most patients in most circumstances without reservation
1C			
Strong recommendation, low-quality, or very low-quality evidence	Benefits clearly outweigh risk and burdens, or vice versa	Observational studies or case series	Strong recommendation but subject to change when higher quality evidence becomes available
2A			
Weak recommendation, high-quality evidence	Benefits closely balanced with risks and burden	RCTs without important limitations or overwhelming evidence from observational studies	Weak recommendation, best action may differ depending on the patient, treatment circumstances, or social values
2B			
Weak recommendation, moderate-quality evidence	Benefits closely balanced with risks and burden	RCTs with important limitations (inconsistent results, methodological flaws, indirect, or imprecise) or exceptionally strong evidence from observational studies	Weak recommendation, best action may differ depending on the patient, treatment circumstances, or social values
2C			
Weak recommendation, low-quality, or very low-quality evidence	Uncertainty in the estimates of benefits, risks, and burden; benefits, risk, and burden may be closely balanced	Observational studies or case series	Very weak recommendation; alternative treatments may be equally reasonable and merit consideration

## Principles of sepsis control

The key factors in the effective treatment of cIAIs are (a) a prompt diagnosis, (b) adequate resuscitation, (c) early initiation of appropriate antibiotic therapy, (d) early and effective source control, and finally, (e) reassessment of the clinical response and appropriate adjustment of the management strategy.

Abdominal sepsis represents the host’s systemic inflammatory response to intra-abdominal infections. Sepsis is a dynamic process that can evolve into conditions of varying severity [10, 11]. The inflammatory response in patients with sepsis depends on the causative pathogen and the host (genetic characteristics and co-existing illnesses), with differential responses at local, regional, and systemic levels [11]. If left untreated, it may lead to the functional impairment of one or more vital organs or systems. It was previously defined that severity of illness and the inherent mortality risk escalate from sepsis, through severe sepsis and septic shock up multi-organ failure. However, differences in the spectrum of etiology and patient factors, including age and co-morbidities, make the course of sepsis different from patient to patient. Illustrating the importance of age, recent data from a consecutive, population-based cohort of patients with perforated gastroduodenal ulcer (PGDU), showed that octa- and nona-genarians with PGDU presented with fewer signs of peritonitis and had an attenuated inflammatory response [12]. HIV patients, common in sub-Saharan Africa, have an increased risk to develop sepsis due to the HIV infection itself that affects several components of the immune system involved in sepsis pathogenesis [13]. HIV causes increased susceptibility to invasive infections and affects sepsis pathogenesis caused by pre-existing activation and exhaustion of the immune system [14], and even if HIV-infected patients on antiretroviral therapy can now safely undergo major abdominal surgery with encouraging results, they are still relatively poorer than those of HIV-negative subjects [15].

Third International Consensus Definitions for Sepsis and Septic Shock (Sepsis-3) has recently been published [16] and updated previous classifications [17, 18]. Sepsis is defined as life-threatening organ dysfunction caused by a dysregulated host response to infection. Organ dysfunction can be represented by an increase in the Sequential (sepsis-related) Organ Failure Assessment (SOFA) score of 2 points or more. Septic shock should be defined as a subset of sepsis and should be clinically identified by a vasopressor requirement to maintain a mean arterial pressure of 65 mm Hg or greater and serum lactate level greater than 2 mmol/L (>18 mg/dL) in the absence of hypovolemia. The definition of severe sepsis is now superfluous. The new definition of sepsis suggests that patients with at least 2 of these 3 clinical variables: Glasgow Coma Scale score of 13 or less, systolic blood pressure of 100 mm Hg or less, and respiratory rate 22/min or greater (quick SOFA - qSOFA) may be prone to a poor outcome typical of sepsis and patients with positive qSOFA should be clinically characterized as septic by SOFA score (Table 2). The SOFA score (Table 2) was proposed in 1996 by the Working Group on Sepsis-Related Problems of the European Society of Intensive Care Medicine [19] to objectively describe the degree of organ dysfunction over time and to evaluate morbidity in intensive care unit (ICU) in patients with sepsis. It was demonstrated to be a good indicator of prognosis in critically ill patients during the first few days of ICU admission [20].

**Table 2:** SOFA Score

PaO_2_/FiO_2_ (mmHg)	SOFA score
<400	1
<300	2
<200 and mechanically ventilated	3
<100 and mechanically ventilated	4
Glasgow coma scale	
13–14	1
10–12	2
6–9	3
<6	4
Mean arterial pressure OR administration of vasopressors required	SOFA score
MAP <70 mm/Hg	1
dop ≤5 or dob (any dose)	2
dop >5 OR epi ≤0.1 OR nor ≤0.1	3
dop >15 OR epi >0.1 OR nor >0.1	4
Bilirubin (mg/dl) [μmol/L]	
1.2–1.9 [>20–32]	1
2.0–5.9 [33–101]	2
6.0–11.9 [102–204]	3
>12.0 [>204]	4
Platelets × 10^3^/μl	
<150	1
<100	2
<50	3
<20	4
Creatinine (mg/dl) [μmol/L] (or urine output)	
1.2–1.9 [110–170]	1
2.0–3.4 [171–298, 305]	2
3.5–4.9 [300–440] (or <500 ml/d)	3
>5.0 [>440] (or <200 ml/d)	4

Some concerns about the new definition of sepsis have been reported [21].

Since the first classification in 1991 [17], the definitions of sepsis, severe sepsis, and septic shock, though imprecise, have provided to clinicians a useful framework for clinical management, stressing the need for early recognition. The new definition of sepsis requiring the presence of organ failure has lost its predictive potential and may hinder the awareness of the importance of early recognition and treatment of sepsis, de-emphasizing intervention at earlier stages when it is most treatable, and leading to a higher risk of delayed diagnosis. Early recognition of sepsis is a general principle of sepsis management and is very important in LMICs where the priorities for improving quality of care of critically ill patients are different.

Documenting the burden of critical illness in low-resource settings is challenging. In these settings, a robust triage system that quickly recognizes critically ill patients and transfers them immediately to an acute care unit are a vital component of the emergency services [22].

Moreover in many areas worldwide, there are limited resources for intensive investigations, as a consequence, any process of improving quality of sepsis care globally should focus on simple diagnostic criteria based on physical examination findings that can recognize patients needing critical care. In these settings, a feasible, low-cost method of rapidly identifying patients requiring critical care should be crucial.

Sepsis-3 definition introduces Quick SOFA (qSOFA) as a tool for identifying patients at risk of sepsis with a higher risk of hospital death both inside and outside critical care units. However, qSOFA does not define sepsis and the new sepsis definitions recommend using an increase in the SOFA score of two points or more to represent organ dysfunction. The SOFA score is potentially not accessible everywhere, especially for PaO_2_, which would require an arterial blood gas measurement.

Early warning scores utilize physiological, easy-to-measure parameters, assessing physiological parameters such as systolic blood pressure, pulse rate, respiratory rate, temperature, oxygen saturations, and level of consciousness [23].

In patients with intra-abdominal infections, early warning scores associated with abdominal signs and symptoms such as abdominal pain and abdominal rigidity can screen patients needing immediate acute care surgery.

Finally, although some patients with ongoing sepsis may not have elevated lactate levels at presentation or during their clinical course [24, 25], lactate measurement is advised as an important component of the initial evaluation of patients with sepsis. Elevated lactate levels (even if >4 mmol/l) are no longer part of organ dysfunction criteria to define sepsis. According to the new definition of sepsis, high lactate levels should be used only as one of the criteria to define septic shock.

*Early recognition of the patient with ongoing abdominal sepsis is an essential step for an effective treatment.*

*Prompt administration of intravenous fluids for resuscitation is critical in patients with an ongoing sepsis. This initial resuscitation should be titrated to the clinical response, and not solely guided by a predetermined protocol. Vasopressor agents may serve to augment and assist fluid resuscitation, particularly where this therapy alone is failing (Recommendation 1A).*

The data from WISS study showed that mortality was significantly affected by sepsis––mortality by sepsis status was no sepsis 1.2%, sepsis only 4.4%, severe sepsis 27.8%, and septic shock 67.8% [1].

Identifying patients with ongoing sepsis early and correcting the underlying microvascular dysfunction may improve patient outcomes. If not corrected, microvascular dysfunction can lead to global tissue hypoxia, direct tissue damage, and ultimately, organ failure [26].

Fluid therapy to improve microvascular blood flow and increase cardiac output is an essential part of the treatment of patients with sepsis. Crystalloid solutions should be the first choice because they are well tolerated and cheap [27]. They should be infused rapidly to induce a quick response but not so fast that an artificial stress response develops. They should be interrupted when no improvement of tissue perfusion occurs in response to volume loading. Basal lung crepitations may indicate fluid overload or impaired cardiac function. Recently, measuring inferior vena cava (IVC) diameter by ultrasound was suggested as a novel outcome measure to guide this resuscitative approach [28].

The Surviving Sepsis Campaign (SSC) is a joint collaboration of the Society of Critical Care Medicine and the European Society of Intensive Care Medicine committed to reducing mortality from severe sepsis and septic shock worldwide in 2002. In 2012, the SSC updated its guidelines. SSC guidelines have been regarded as the standard of care in patients with severe sepsis and septic shock in many hospitals worldwide [29]. However, the possibility to implement the SSC guidelines has been questioned in LMICs where simple and low-cost standardized laboratory testing should be emphasized to allow accurate diagnosis, prognosis, and monitoring of treatment response [30, 31]. A study conducted as an anonymous questionnaire-based, cross-sectional survey among anaesthesia providers, suggested that the most recent SSC guidelines cannot be implemented in Africa, particularly in Sub-Sahara Africa, due to a shortage of required hospital facilities, equipment, drugs, and disposable materials [32].

The 2016 Surviving Sepsis Campaign International Guidelines for Management of Sepsis and Septic Shock was recently published [33]. Previous iterations of these guidelines aimed to treat the early hypovolemic phase of sepsis by providing appropriate high volume resuscitation targeting central venous pressure 8–12 mm Hg, mean arterial pressure (MAP) >65 mm Hg, urine output >0.5 mL/kg/h, central venous (superior vena cava) or mixed venous oxygen saturation >70 or >65%, respectively. Since the first draft of guidelines, the basic concept of the initial resuscitation has been early goal-directed therapy (EGDT) described by Rivers in 2001, who reported that patients with severe sepsis and septic shock presenting to the emergency department had a lower mortality rate, if they received a specific 6-h resuscitation bundle of EGDT [34]. Recent randomized controlled trials (ProCESS, ARISE, and ProMISe trials) results [35–37] have questioned River’s resuscitation protocol results demonstrating that use of early goal-directed therapy for patients presenting to the emergency department with early septic shock did not reduce mortality compared with usual care.

These data indicate that an early identification and prompt administration of intravenous fluids are mandatory. However, initial resuscitation should no longer be based on a predetermined protocol but on clinical endpoints.

Hypotension is the most common indicator of inadequate perfusion. The SSC advocated a mean arterial pressure (MAP) goal of 65 mm Hg during the first 6 h of treatment. It was confirmed by a randomized controlled trial “Sepsis and Mean Arterial Pressure” (SEPSISPAM) examining high versus low MAP goals in patients with septic shock. It demonstrated that targeting a mean arterial pressure of 80–85 mm Hg, as compared with 65–70 mm Hg, in patients with septic shock undergoing resuscitation did not result in significant differences in mortality at either 28 or 90 days [38].

Particularly in patients with abdominal sepsis, requiring urgent surgical intervention, overly aggressive fluid resuscitation may increase intra-abdominal pressure and worsen the inflammatory response, which is associated with a high risk of complications [39, 40]. In patients with septic shock fluid infusion during resuscitation, bowel oedema and forced closure of the abdominal wall can cause intra-abdominal hypertension and abdominal compartment syndrome that can consequently modify pulmonary, cardiovascular, renal, splanchnic, and central nervous system physiology causing significant morbidity and mortality. Clinical endpoints in monitoring fluid volume infusions should include mean arterial pressure, skin color and capillary refill, mental status, or urinary output. Central venous access, where available, may be helpful for monitoring of central venous pressure. Simpler non-invasive devices such as tissue perfusion monitors may be more practical but are not yet widely used. Repeated measurements of IVC diameter by ultrasound can be a simple and useful method for defining fluid requirements [28].

Vasopressor agents should be administered to restore organ perfusion if fluid resuscitation fails optimizing blood flow and if hypotension persists following fluid loading. These agents should be globally available. Vasopressor and inotropic agents have increasingly become a therapeutic cornerstone for the management of sepsis. They have excitatory and inhibitory actions on the heart and vascular smooth muscle, as well as important metabolic, central nervous system, and presynaptic autonomic nervous system effects. The optimal timing of vasopressors relative to fluid infusion has been debated. Recently, a large multi-center retrospective analysis of 2849 patients with septic shock, investigators found that mortality was lowest when vasopressors were delayed by 1 h and infused from hours 1 to 6 following onset of shock [41]. Norepinephrine is now the first-line vasopressor agent used to correct hypotension in the event of septic shock [29]. Norepinephrine is more efficacious than dopamine and may be more effective for reversing hypotension in patients with septic shock [29]. Dopamine may cause more tachycardia and may be more arrhythmogenic than norepinephrine, and as an alternative vasopressor agent to norepinephrine, it should be used only in patients with low risk of tachyarrhythmias and absolute or relative bradycardia.

Dobutamine is an inotropic agent used to treat septic shock patients increasing cardiac output, stroke index, and oxygen delivery (DO_2_). It has been suggested to be administered to pre-existing vasopressor therapy in the presence of myocardial dysfunction, defined as elevated cardiac filling pressures and low cardiac output. However, dobutamine increases DO_2_ to supranormal values and in critically ill patients, it has raised serious questions regarding its safety in the treatment of septic shock. Because dobutamine provides direct stimulation of the β-1 adrenergic receptors, it is recognized as more problematic with regard to tachycardia and arrhythmia.

In LMICs, it may be acceptable to use adrenaline infusions as the inotrope of choice, given it is readily available, cheap, and has been shown to be equivalent to noradrenaline in septic shock [42].

Increased global availability of vasopressors together with a better understanding of their indications, pharmacodynamics, and important adverse effects are mandatory to fight sepsis worldwide.

## Diagnosis

*A step-up approach for diagnosis from clinical and laboratory examination*, *to imaging examination should be used and tailored to the hospitals resources* (*Recommendation 1B*).

The diagnosis of intra-abdominal infections is based primarily on clinical assessment. Typically, the patient is admitted to the emergency department with abdominal pain and a systemic inflammatory response, including fever, tachycardia, and tachypnoea. Abdominal rigidity suggests the presence of peritonitis. Hypotension and hypoperfusion signs such as lactic acidosis, oliguria, and acute alteration of mental status are indicative of ongoing sepsis.

In many countries worldwide, a large proportion of patients with diffuse peritonitis still present to the hospital with unacceptable delay. This event reduces the percentage of surviving at the lowest rates in the world [8]. In emergency departments of limited-resource hospitals, the diagnosis of intra-abdominal infections is mainly clinical; supported by basic laboratory tests like full blood count (complete blood count). Ultrasonography is sometimes done to aid diagnosis, if available. Therefore, the clinician has to improve clinical diagnosis by looking carefully for those signs and symptoms. In rural and remote areas of LMICs, diagnostic imaging is often insufficient, and in some instances, completely lacking [43]. In recent years, ultrasound use has increased worldwide, facilitated by ultrasound machines becoming smaller, more reliable, and less expensive [44]. Ultrasound is reproducible and can be easily repeated, but remains highly user-dependent, and thus, experience should be taken into account for diagnostic accuracy and reliability.

In rural areas of LMICs with limited access to surgical care and CT, plain X-ray abdomen and ultrasound can help to identify and diagnose surgical emergencies cost-effectively, allowing efficient use of resources [45, 46].

CT may be very useful especially when the diagnosis is uncertain. In high-income countries, it has become the gold standard. In 2006, a meta-analysis by Doria et al. demonstrated that CT imaging featured significantly higher sensitivity and resolution than ultrasound in studies of both children and adults with acute appendicitis [47].

Proposals of staged algorithmics with a step-up approach with CT performed after an inconclusive or negative US were proposed in the setting of acute appendicitis and acute diverticulitis [48–51].

## Source control

The timing and adequacy of source control are important in the management of intra-abdominal infections; late and/or incomplete procedures may have severely adverse consequences on outcome especially in critically ill patients.

IAIs include several different pathological conditions and are usually classified into uncomplicated and complicated [52]. In uncomplicated IAIs, the infectious process only involves a single organ and does not proceed to peritoneum. Patients with such infections can be managed with either surgical source control or with antibiotics alone. In complicated IAIs (cIAIs), the infectious process extends beyond the organ and causes either localized peritonitis or diffuse peritonitis. The treatment of patients with complicated intra-abdominal infections involves both source control and antibiotic therapy.

Peritonitis is classified into primary, secondary, or tertiary peritonitis [52]. Primary peritonitis is a diffuse bacterial infection without loss of integrity of the gastrointestinal tract in absence of an identifiable source of infection during surgical exploration; this is rare, and mainly occurs in infancy and early childhood as well as in cirrhotic patients. Secondary peritonitis, the most common form of peritonitis, is an acute peritoneal infection resulting from loss of integrity of the gastrointestinal tract or from infected viscera. It is caused by perforation of the gastrointestinal tract (e.g., perforated duodenal ulcer) by direct invasion from infected intra-abdominal viscera (e.g., gangrenous appendicitis). Anastomotic dehiscences are common causes of secondary peritonitis in the postoperative period. Tertiary peritonitis is a recurrent infection of the peritoneal cavity that follows either primary or secondary peritonitis. It is a complication of a secondary peritonitis and may be termed also “ongoing peritonitis” or “persistent” peritonitis [53, 54].

As a general principle, the most established source of infection should be totally controlled as soon as possible. However, source control requires general anaesthesia that may not be readily available in some areas of the world. In many rural areas, patients must be centralized to urban centers with the loss of much time, largely due to transport delays. Furthermore, the urgency of intervention is also affected by the rapidity of the evolution of clinical symptoms.

Source control encompasses all measures undertaken to eliminate the source of infection, reduce the bacterial inoculum, and correct or control anatomic derangements to restore normal physiologic function [55, 56]. The primary objectives of intervention include (a) determining the cause of peritonitis, (b) draining fluid collections, and (c) controlling the origin of the abdominal sepsis. This endeavor generally involves drainage of abscesses or infected fluid collections, debridement of necrotic or infected tissues, and definitive control of the source of contamination. Control of the septic source can be achieved either by surgical or non-surgical means. Non-surgical interventional procedures imply percutaneous drainages of abscesses, when it is available. Ultrasound and CT-guided percutaneous drainage of abdominal and extraperitoneal abscesses in selected patients are safe and effective [57–61]. Surgical source control includes resection or suture of a diseased or perforated viscus (e.g., diverticular perforation, gastroduodenal perforation), removal of the infected organ (e.g., appendix, gallbladder), debridement of necrotic tissue, resection of ischemic bowel, and repair/resection of traumatic perforations with primary anastomosis or exteriorization of the bowel.

In recent years, laparoscopy has been gaining wider acceptance in the diagnosis and treatment of intra-abdominal infections. The laparoscopic approach in the treatment of peritonitis is feasible for many emergency conditions. It has the advantage to allow, at the same time, an adequate diagnosis and appropriate treatment with a less invasive abdominal approach [62]. However, laparoscopy due to the increase of intra-abdominal pressure due to pneumoperitoneum, may have a negative effect in critically ill patients leading to acid–base balance disturbances, as well as changes in cardiovascular and pulmonary physiology [40]. Laparoscopy is still uncommon in many areas of the world for several reasons, a major one being cost. In these countries, the challenges posed by the burden of primary healthcare concerns have limited government support for development of modern tertiary healthcare facilities, and laparoscopic surgery is practiced in only a few tertiary hospitals. Innovative programs to train surgeons and develop low-cost equipment in these countries are encouraging [63]. Some studies in the literature have focused on the feasibility of implementing laparoscopic procedures in resource-poor countries, and how to overcome the challenges involved [64–66].

Etiological factors of peritonitis show a wide geographical variation and different spectrum in the various regions of the world. Table 3 summarizes the sources of infection in the recent international WISS Study [1].

**Table 3:** Source of infection in 4553 patients from 132 hospitals worldwide (15 October 2014–2015 February 2015) [1]

Source of infection	Number (%)
Appendicitis	1553 (34.2)
Cholecystitis	837 (18.5)
Post-operative	387 (8.5)
Colonic non-diverticular perforation	269 (5.9)
Gastro-duodenal perforations	498 (11)
Diverticulitis	234 (5.2)
Small bowel perforation	243 (5.4)
Others	348 (7.7)
PID	50 (1.1)
Post traumatic perforation	114 (2.5)
Total	4553 (100)

## Acute appendicitis

Acute appendicitis is both the most common general surgery emergency presentation, as well as the most common cause of intra-abdominal sepsis, worldwide. The WISS study [1] confirmed acute appendicitis as the most frequent cause of intra-abdominal sepsis and demonstrated that around one-third of these cases were complicated. Interestingly, the incidence of acute appendicitis varies: it is generally thought to have a low-incidence rate in sub-Saharan Africa and in many regions of Asia and Latin America. This condition once thought to be rare in many regions of the world, but appears to be increasing in many urban centers and also in LMICs, perhaps due to changes in life style and diet [65]. However, the true incidence of appendicitis in many areas of the world is unknown due to poor medical record-keeping and unreliable population census. In 2015, a retrospective study over a 4-year period in South Africa [66] reported over half (56%) were from the urban district within the city of Pietermaritzburg and the remaining 44% were from the rural health district. The median duration of illness from onset to definitive care was 4 days. Sixty percent of appendices were perforated and associated with intra-abdominal contamination. Forty percent of patients required reoperation to control intra-abdominal sepsis. Ten percent required admission to the intensive care unit. The median overall length of hospital stay was 5 days. The mortality rate was 1%. Rural patients had a longer median duration of illness (3 versus 5 days, *p* < 0.001) as well as a more advanced disease profile associated with perforation and severe intra-abdominal sepsis (19 versus 71%, *p* < 0.001).

The natural history of appendicitis has been described in three stages: (1) a normal appendix, (2) uncomplicated acute appendicitis, and (3) complicated appendicitis, according to their macroscopic and microscopic appearance and clinical relevance [67]. The high morbidity and occasional mortality associated with acute appendicitis are related to delay in presentation by patients or delay in diagnosis by the clinician. These delays may result in complications like gangrene, perforation, appendiceal mass, and peritonitis, all of which would prolong hospital stay and increase the cost of treatment.

Unfortunately, the clinical presentation of appendicitis is often inconsistent. While the clinical diagnosis may be clear in patients presenting with classic signs and symptoms, atypical presentations may result in delay in treatment. Therefore, diagnostic scoring systems have been described with the aim to provide clinical probabilities that a patient has acute appendicitis. The development of these scores may contribute to diagnosis and by easily applicable clinical criteria and simple laboratory tests a score which classifies the probability of diagnosis may be attributed to the patient. In 1986, Alvarado published his own method for the early diagnosis of acute appendicitis [68]. A score of five or six was compatible with the diagnosis of acute appendicitis, a score of seven or eight indicated a probable appendicitis, and a score of nine or ten indicated a very probable appendicitis. The only laboratory tests needed in the initial evaluation for acute appendicitis was a complete blood count to determine if there was shift to the left or increased segmented neutrophils (more than 75%) [69]. The more recently introduced appendicitis inflammatory response (AIR) score incorporated the C-reactive protein value in its design and was developed and validated on a prospective cohort of patients with suspicion of acute appendicitis. It was based on similar values to the Alvarado score, but it also included C-reactive protein as a new variable [70].

Recently, WSES guidelines for diagnosis and treatment of acute appendicitis were published [71]

*Appendectomy remains the treatment of choice also for acute appendicitis. Antibiotic therapy is a safe means of primary treatment for patients with uncomplicated acute appendicitis, but it is less effective in the long-term due to significant recurrence rates and probably needs the certainty of a CT proven diagnosis of uncomplicated appendicitis* (*Recommendation 1A*).

Antibiotics alone may be useful to treat patients with early, non-perforated appendicitis, even if there is a risk of recurrence [71, 72]. In the APPAC (Antibiotic Therapy versus Appendectomy for Treatment of Uncomplicated Acute Appendicitis) trial recently published in JAMA [73] enrolling 530 patients with uncomplicated appendicitis confirmed by a CT-scan (257 antibiotic therapy, 273 appendectomy), the 1-year recurrence rate and appendectomy in the antibiotic group was reported as 27%. Although antibiotic therapy can be successful in selected patients with uncomplicated appendicitis, the risk of disease recurrence limits the application of this treatment strategy. Apart from this high recurrence rate, the need for additional diagnostic certainty with a CT-proven diagnosis further complicates this approach [74]. Finally, in this era of antimicrobial resistance, antibiotic overuse should be limited. For all these reasons, appendectomy has remained in international guidelines the gold-standard treatment for acute appendicitis worldwide [75].

*Both open and laparoscopic appendectomies are viable approaches to surgical treatment of acute appendicitis (Recommendation 1A).*

The advent of laparoscopy has modified the surgical treatment of acute appendicitis in high-income countries. In contrast, in many areas of the world, the challenges posed by the burden of primary healthcare concerns have limited support for development of modern tertiary healthcare facilities, and laparoscopic surgery is practiced in only a few tertiary hospitals. In the last years, several prospective randomized studies, meta-analyses, and systematic critical reviews have been published on the topics of laparoscopic appendectomy. Laparoscopic appendectomy is safe and effective, but open surgery still confers benefits, in particular with regard to the likelihood of postoperative intra-abdominal abscess. In a meta-analysis by Li et al. [76] including 44 randomized controlled trials with 5292 patients laparoscopic appendectomy (LA) provided considerable benefits over open appendectomy (OA), including a shorter length of hospital stay, less postoperative pain, earlier postoperative recovery, and a lower complication rate. However, LA was associated with a slight increase in the incidence of intra-abdominal abscess, intra-operative bleeding and urinary tract infection. Sauerland et al. [77] performed a meta-analysis including 67 studies, of which 56 compared LA (with or without diagnostic laparoscopy) versus OA in adults wound infections were less likely after LA than after OA, but the incidence of intra-abdominal abscesses was increased. In a prospective study published in 2010, Tzovaras et al. found that the postoperative length of hospital stay did not differ significantly between OA and LA for men. Laparoscopic appendectomy required more time and did not offer any advantages compared with OA in men [78].

*Patients with a periappendiceal abscess can be managed with percutaneous image-guided drainage in surgical departments with ready access to diagnostic and interventional radiology. When percutaneous drainage is not available surgery is suggested (Recommendation 1B).*

In approximately 10% of patients, a periappendiceal abscess and inflammatory phlegmon is present at diagnosis. This is more frequently encountered in the situation of a delayed diagnosis. Clinical features of complicated appendicitis such as mass and abscess may include fever, tachycardia, palpation of mass, and extension of area of tenderness and rebound. Surgical management varies because a proportion of these cases will evolve into an ileocecal resection or a right-sided hemicolectomy if operated in the acute setting [79]. In recent years, high success rates of 76–97% [80] and low incidences of complications have been reported in patients with appendicitis associated with abscess and/or mass, after conservative management; thus, performing non-surgical treatments, such as antibiotic therapy and percutaneous drainage, during the initial period have been proven to be effective and safe [81–84]. However, a necessary condition for conservative management of these patients is an easy access to diagnostic and interventional radiology to perform a percutaneous drainage. When percutaneous drainage is not available, surgery is suggested [85, 86].

*In patients treated conservatively, interval appendectomy may be not necessary following initial non-operative treatment of complicated appendicitis. However, interval appendectomies should always be performed for patients with recurrent symptoms (Recommendation 2B)* ***.***

Traditionally, an interval appendectomy has been offered to patients who initially underwent a non-operative approach to their appendiceal mass. However, the role of the interval appendectomy has been questioned, and controversy continues whether interval appendectomy is appropriate for adults with an appendiceal abscess. The main debate revolves around the recurrence rate, the complication rate of an interval appendectomy, and the potential for underlying malignancy. The results of a review by Andersonn and Petzold, based mainly on retrospective studies, supported the practice of non-surgical treatment without interval appendectomy in patients with appendiceal abscess or phlegmon [81]. However, the patient should be informed about the risk of recurrence especially in the presence of appendicolith. The risk of missing another underlying condition (cancer or Crohn disease) is low, but motivates a colonoscopy in patients above the age of 40 years.

*Routine use of intraoperative irrigation for appendectomies does not prevent intra-abdominal abscess formation and may be avoided (Recommendation 2B).*

In 2011, a retrospective review of 176 consecutive appendectomies, open (39%) and laparoscopic (61%), at a university-affiliated tertiary care facility from July 2007 to November 2008 investigated routine use of intra-operative irrigation for appendectomies. The results did not show a decrease in postoperative intra-abdominal abscess with the use of intra-operative irrigation. Thirteen patients developed postoperative abscess: 11 with irrigation and two without irrigation. Ten of 13 patients who developed abscess were perforated; nine with irrigation and one without [87].

## Acute left colonic diverticulitis

Acute sigmoid diverticulitis is a common disease of the Western World and results in a significant number of hospital admissions. Data from Western populations suggest that up to one fifth of patients with acute diverticulitis are under the age of 50 years of age [88, 89]. Recent evidence suggests that lifetime risk of developing acute left-sided colonic diverticulitis (ALCD) is only about 4% among patients with diverticulosis [90].

Recent WSES guidelines for the management of acute diverticulitis in the emergency setting were published [91].

Clinical findings of patients having ALCD include acute pain or tenderness in the left lower quadrant, which may be associated with increased inflammatory markers including C-reactive protein (CRP) and white blood cell count (WBC). However, the clinical diagnosis of ALCD lacks accuracy: in a prospective analysis [92] conducted on 802 consecutive patients that presented with abdominal pain to the emergency department, positive and negative predictive values of clinical diagnosis were 0.65 and 0.98, respectively. Additional cross-sectional imaging had a positive and negative predictive value of more than 0.95 and 0.99, respectively. Radiology examinations improved the diagnostic accuracy in 37% of the patients, although only changed the management in 7%.

*Antibiotics can be avoided in patients with CT findings of uncomplicated ALCD and without significant comorbid conditions or signs of sepsis. Patients should be clinically monitored to assess for resolution of the inflammatory processes (Recommendation 1A).*

ALCD is generally divided into uncomplicated and complicated. The utility of antimicrobial therapy in acute uncomplicated diverticulitis has been a point of controversy in the international medical community [93]. In terms of clinical resolution, recent investigation demonstrates that antimicrobial treatment was not superior to withholding antibiotic therapy in patients with mild, unperforated diverticulitis. Furthermore, a multi-center randomized trial involving ten surgical departments in Sweden and one in Iceland, recruited 623 patients with computed tomography-verified acute uncomplicated left-sided diverticulitis [94], and concluded that antibiotic treatment for acute uncomplicated diverticulitis neither accelerated recovery nor prevented complications or recurrence. In these studies, the definition of uncomplicated ALCD is based on strict CT scan definitions: for example, patients with pericolic free gas or even minimal free fluid were classified as having complicated disease and were excluded from investigation [95].

*On the basis of clinical conditions, patients with diverticular smaller abscesses may be treated by antibiotics alone (Recommendation 1C).*

*Patients with abscesses having a large diameter should be treated by percutaneous drainage and intravenous antibiotics (Recommendation 1C).*

*Whenever percutaneous drainage of the abscess is not feasible or not available, based on the clinical conditions patients with large abscesses can be initially treated by antibiotic therapy alone. However careful clinical monitoring is mandatory (Recommendation 1C).*

Approximately 15–20% of patients admitted with ALCD have an abscess [96], and the treatment should be either antibiotics with or without percutaneous and/or surgical drainage. The use of antibiotics and percutaneous drainage in the management of diverticular abscesses facilitates single-stage operation to perform subsequently an elective sigmoidectomy. The size of 3–6 cm has been generally accepted, despite the low level of evidence, to be a reasonable limit between antimicrobial versus percutaneous drainage in the management of diverticular abscesses [96–100].

A retrospective study assessing the effectiveness of antibiotics as sole initial therapy in patients with large diverticular abscess was published in 2015 by Elagili et al. [101]. Thirty-two patients were managed by antibiotics alone while 114 underwent percutaneous drainage.

Failure of initial treatment required urgent surgery in eight patients with persistent symptoms during treatment with antibiotics alone (25%) and in 21 patients (18%) after initial percutaneous drainage (*p* = 0.21). Patients treated with antibiotics had a significantly smaller abscess diameter (5.9 versus 7.1 cm, *p* = 0.001). Postoperative complications in patients treated with antibiotics alone were significantly less severe than after percutaneous drainage based on the Clavien-Dindo classification (*p* = 0.04).

*Hartmann’s procedure remains useful in the management of diffuse peritonitis in critically ill patients. However, in clinically stable patients, primary resection with anastomosis, with or without a diverting stoma, may be performed (Recommendation 1B).*

Hartmann’s procedure has been considered the procedure of choice in patients with generalized peritonitis and remains a safe technique for emergency colectomy in diverticular peritonitis, especially in critically ill patients and in patients with multiple co-morbidities. However, restoration of bowel continuity after Hartmann’s procedure has been associated with significant morbidity [102]. In recent years, some authors have reported the role of primary resection and anastomosis with or without a diverting stoma in stable patients without comorbidity, even in the presence of diffuse peritonitis [103]. Studies comparing mortality and morbidity of Hartmann’s procedure versus primary anastomosis did not show any significant differences. However, most studies had relevant selection bias as demonstrated by four systematic reviews [103–107].

*Laparoscopic peritoneal lavage and drainage may not be considered the treatment of choice in patients with diffuse peritonitis (Recommendation 1A).*

Recent investigation continues to define the role of laparoscopic peritoneal lavage in the treatment of ALCD, and unanswered questions remain. The latest prospective trials, including the SCANDIV, Ladies, and DILALA trials [108–111], have lacked superiority for lavage in terms of morbidity, but mortality was not compromised. A meta-analysis published in 2015 showed that in acute perforated diverticulitis with purulent peritonitis laparoscopic lavage is comparable to sigmoid resection in terms of mortality, but it is associated with a significantly higher rate of reoperations and a higher rate of intra-abdominal abscess [111]. No differences in terms of mortality were demonstrated at follow-up.

## Colonic carcinoma perforation

*Treatments for perforated colonic carcinoma should both stabilize the emergency condition of the peritonitis and fulfil the technical objectives of oncological intervention (Recommendation 1B).*

Patients with perforated colonic carcinoma had a significantly poorer prognosis compared with patients with non-perforated colonic cancer. Colorectal cancer-induced perforation is considered an advanced stage disease due to the potential for peritoneal dissemination of tumor cells throughout the site of perforation [112, 113].

The stage of illness, proximity of the perforation to the tumor, and the number of metastatic lymph nodes are positively correlated with reduced procedural- and cancer-free survival rates [114].

Hartmann’s procedure has been widely accepted as an effective means of treating carcinoma of the left colon (with adequate R0 resection) in certain emergency scenarios [115].

## Colonic perforation following colonoscopy

*Patients presenting with diffuse peritonitis caused by colonoscopic perforation should undergo immediate surgical intervention, which typically involves primary repair or resection (Recommendation 1B).*

Recently, the frequency of colonic perforation has increased due to routinely performed advanced therapeutic endoscopy. The recent advent of endoscopic submucosal dissection (ESD) has resulted in a high incidence of perforation, although the indication for endoscopic therapy of colorectal neoplasm has been expanded.

Over the last decade, many advancements have been streamlined to better address these perforations, yet there are no definitive guidelines for their optimal management [116].

Endoscopic management is typically used to treat colonoscopy-associated perforations if they can be closed by endoscopic clipping during colonoscopy [117–119].

Retrospective studies showed that conservative management of colonoscopic perforation could be an option for patients without overt symptoms of peritonitis or with a small defect size [120, 121].

Early exploratory laparotomy with primary closure or bowel resection has been the standard treatment of colonoscopic perforation [122]. In cases of extensive contamination, poor tissue quality and a higher complication rate, stoma, or fecal diversion after repair should be performed [123].

Iqbal et al. in a retrospective study [124] indicated that factors predicting a poor outcome included delayed diagnosis, extensive peritoneal contamination, and patients using anticoagulants (*p* < .05).

*An early laparoscopic approach may be a safe and effective option for colonoscopy-related colonic perforation for experienced surgeons (Recommendation 2B).*

Laparoscopic surgery is a compromise that may minimize the risks of invasive surgery as well as those of insufficiently aggressive non-operative therapy [125–128].

In the Zhang et al. study, their experience in laparoscopic direct suturing of colon perforations indicated that laparoscopic primary perforation repair was a safe and feasible repair method [127].

If the area of perforation cannot be localized laparoscopically, the surgeon should begin with a laparotomy before proceeding further [128].

## Gastroduodenal peptic ulcer perforations

Gastroduodenal ulcer perforations have decreased in the last few years, largely due to the widespread adoption of medical therapies for peptic ulcer disease and decreasing incidence of helicobacter pylori infection in Western countries. However, ulcer disease is a still common emergency condition worldwide and is associated with mortality rates of up to 30% [129, 130]. The main etiologic factors include use of non-stereoidal anti-inflammatory drugs (NSAIDs), steroids, smoking, *Helicobacter pylori* (*H. pylori*) and a diet high in salt. All these factors have in common that they affect acid secretion in the gastric mucosa [129]. Stress ulcers with perforation may occur in critically ill patients in intensive care, where the diagnosis may be obscured owing to lack of signs and symptoms in an unconscious or sedated patient.

*Surgery is the treatment of choice for perforated peptic ulcers (Recommendation 1A).*

*Simple closure with or without an omental patch is a safe and effective procedure to address small perforated ulcers (<2 cm) (Recommendation 1A).*

Surgery is the most effective means of source control in patients with PPU [131]. The main surgical treatment for PPU has become simple suture of the perforation site with or without the addition of an omental patch [132].

In 2010, Lo et al. conducted a study to determine if an omental patch offers any clinical benefit that is not offered by simple closure alone [133]. The study demonstrated that, in terms of leakage rates and overall surgical outcome, covering the repaired perforated peptic ulcer with an omental patch did not convey additional advantages compared to simple closure alone.

Scoring systems to predict disease severity or outcome in patients with gastroduodenal perforations are unreliable and not accurate and cannot be generalized from one population to another [133, 134].

*Laparoscopic repair of perforated peptic ulcers can be a safe and effective procedure for experienced surgeons (Recommendation 1A).*

Successful laparoscopic repair of perforated gastric and duodenal ulcers has been reported, though the technique has yet to be universally accepted. The literature was summarized in a recent systematic review [135]. The authors concluded that laparoscopic surgery results are not clinically different from those of open surgery. Further data is required to investigate the potentially long learning curve seen among participating surgeons.

Conservative treatment for PPU is seldom reported and restricted mostly to case reports and series, [136].

## Small bowel perforations

Small bowel perforations are a less common source of peritonitis in the Western countries, compared with LMICs. In Western countries, most small intestinal perforations are due to unrecognized intestinal ischemia (mesenteric or strangulation) or inflammatory bowel disease such as Crohn disease. This pattern of disease is quite different to LMICs, where small bowel perforations are usually due to typhoid fever. Typhoid fever remains endemic in Asia, Africa, Latin America, the Caribbean, and Oceania [137]. Ileal perforation as complication of typhoid fever and enteritis is a major public health problem in many areas worldwide because of its persistent high morbidity and mortality. Typhoid ileal perforations have a mortality rate up to 60% [138]. In the CIAOW study, according to stepwise multivariate analysis, the presence of small bowel perforation was an independent variable predictive of mortality [139]. The most common clinical presentation of enteric perforation is abdominal pain and fever whereas perforation typically occurs in the third week of disease. Lack of an incidence database and poor financial resources preclude adequate prevention of this public health menace [140]. The preoperative diagnosis of perforation usually is based on findings of peritonitis in a patient with a history of prolonged febrile illness. In a prospective study, 53 consecutive patients with typhoid perforation were surgically treated; the morbidity rate for this series of procedures was 49.1%, and the most common post-operative complications included wound infection, wound dehiscence, burst abdomen, residual intra-abdominal abscesses, and entero-cutaneous fistulae. The mortality rate was 15.1% and was significantly affected by the presence of multiple perforations, severe peritoneal contamination, and burst abdomen [141].

*Surgery is the treatment of choice for patients with small bowel perforations (Recommendation 1B).*

*In the event of small perforations, primary repair is recommended (Recommendation 1B).*

There are many methods of surgical treatment of small bowel perforation, including primary closure, excision and closure, resection and primary anastomosis, limited right hemicolectomy, and stoma creation [142]. Primary repair should be performed for patients with minor symptoms and with perioperative findings of minimal peritoneal contamination of the peritoneal cavity [75]. In the setting of typhoidal perforation, although closure in two layers of single perforation with relatively healthy tissue after refreshment of the edge seems an acceptable option [143], resection of the unhealthy tissue segment with primary anastomosis of healthy edges about 10 cm on each side of the perforation is recommended [144, 145].

In delayed cases with diffuse peritonitis, there can be severe inflammation and oedema of the bowel, resulting in friable tissue which precludes anastomosis, and therefore, an ileostomy should be performed as a life saving measure [75]. Laparoscopic management of small bowel perforations was reported, but there was no comparative study with open surgery [146].

Other infections that may rarely cause small bowel perforation in immuno-compromised patients include amoebic infection, *Clostridium difficile*, *Cytomegalovirus*, and Histoplasmosis [147–150]. Rarely, medications (NSAIDs, potassium chloride, and steroids), cancer chemotherapy and radiotherapy may lead to small bowel perforation.

## Abdominal tuberculosis

Tuberculosis (TB) remains prevalent worldwide. It is considered as a global health problem by the World Health Organization and is considered the most important communicable disease in the world.

Although much of the burden is concentrated in high-burden settings in Asia and Africa, TB continues to be of concern in high-income nations. The number of tuberculosis cases has also been increasing in high-income countries, mainly because of immigration and as a consequence of acquired immunodeficiency syndrome (AIDS) and also because of the utilization of immunosuppressive drugs [151].

Abdominal involvement in tuberculosis is the most common extra-pulmonary form of this infection.

The most common site of extra pulmonary tuberculosis is the ileocecal region and terminal ileum [151].

The clinical presentation of tuberculosis is variable and non-specific, with non-pathognomonic signs and symptoms. It may mimic other infectious or inflammatory pathological diseases, and even neoplastic conditions [151, 152].

The most common complication of small bowel tuberculosis is obstruction due to the narrowing of the lumen by ileocecal tuberculosis or stricture of small intestine and perforation in ulcerative type of tuberculosis.

*In the case of abdominal tuberculosis perforation resection of the affected area and anastomosis may be the treatment of choice rather than primary closure (Recommendation 1C).*

Treatment of tubercular perforation of ileum depends upon the condition of the gut, general condition of the patient and number of perforations. The resection of the affected area and anastomosis may be the treatment of choice rather than primary closure [152].

## Acute calculous cholecystitis

Cholelithiasis is a common disorder all over the world [153–155]. Its prevalence varies widely by region: in Western countries, the prevalence of gallstone disease reportedly ranges from approximately 7.9% in men to 16.6% in women [155]; in Asia, it ranges from approximately 3 to 15%, is nearly non-existent (less than 5%) in Africa [156], and ranges from 4.21 to 11% in China.

Acute cholecystitis develops in 1–3% of patients with symptomatic gallstones [157].

In 2016, WSES guidelines for the management of acute calculous cholecystitis (AAC) were published [153].

The diagnosis of acute cholecystitis is made on the basis of clinical features such as right upper quadrant pain, fever, and leukocytosis and is supported by findings from relevant imaging studies. Ultrasound is the investigation of choice in patients suspected of having acute cholecystitis [158]. Ultrasound typically shows pericholecystic fluid (fluid around the gall bladder), distended gall bladder, oedematous gallbladder wall, and gall stones, and Murphy’s sign can be elicited on ultrasound examination [158]. Treatment is predominantly surgical, although the timing of surgery without evidence of gangrene or perforation has been under debate in recent years. Two approaches are available for the treatment of acute cholecystitis: the early option, generally within 7 days of onset of symptoms, offers a laparoscopic cholecystectomy (LC) to provide immediate, definitive surgical treatment after establishing diagnosis and surgical fitness of the patient in the same hospital admission, while the delayed treatment option is performed in a second hospital admission after an interval of 6–12 weeks during which time the acute inflammation settles [159].

*Early cholecystectomy is a safe treatment for acute cholecystitis and generally results in shorter recovery time and hospitalization compared to delayed cholecystectomies (Recommendation 1A).*

Several randomized controlled trials have investigated early laparoscopic cholecystectomy (ELC) versus delayed laparoscopic cholecystectomy (DLC) and meta-analysis [160–168]. A recent meta-analysis comparing early versus delayed laparoscopic cholecystectomy for acute cholecystitis [169] reported on 16 studies, involving 1625 patients: for patients with acute cholecystitis, ELC appears as safe and effective as DLC. ELC might be associated with lower hospital costs, fewer work days lost, and greater patient satisfaction.

Among patients with uncomplicated cholecystitis, if source control is complete, no postoperative antimicrobial therapy is necessary [170].

*Laparoscopic cholecystectomy is a safe and effective treatment for acute cholecystitis (Recommendation 1A).*

*It is the first choice for patients with acute cholecystitis where adequate resources and skill are available. Some risk factors may predict the risk for conversion to open cholecystectomy.*

Multiple prospective trials have demonstrated that the laparoscopic cholecystectomy is a safe and effective treatment for acute cholecystitis [171–174]. As a result, immediate laparoscopic cholecystectomy has largely become the therapy of choice for acute cholecystitis in operable patients. While the laparoscopic approach is usual, several risk factors predicting the need to convert to an open approach are reported. In an evaluation of preoperative risk factors as markers for conversion, a meta-analysis summarizing 11 nRCTs containing 14,645 patients, reported age >65 years, male gender, acute cholecystitis, thickened gallbladder wall, diabetes mellitus, and previous upper abdominal surgery all as significant risks, associated with increased risk of conversion [175, 176]. However, open cholecystectomy remains a feasible option, particularly in low-income countries [177], or elsewhere in the setting of resource limitations. The CIAOW study showed that open cholecystectomy, among the patients with complicated cholecystitis, was the most frequently performed procedure [139].

In LMICs, laparoscopic surgery is just evolving in tertiary centers. Despite the low volume of patients and the absence of fluoroscopy in many hospitals results in treating acute cholecystitis seem to be comparable with high volume centers [177].

*Cholecystostomy is a safe and effective treatment for acute cholecystitis in critically ill and/or with multiple comorbidities and unfit for surgery patients (Recommendation 1B).*

Acute cholecystitis in elderly, critically ill patients still today remains a real challenge to treat. Despite the low rate of surgical impact from the laparoscopic approach, many patients are unfit for any surgery. In this subgroup of patients, urgent cholecystostomy with or without delayed laparoscopic cholecystectomy appears to be the correct clinical approach [178–198].

*Early diagnosis of gallbladder perforation and immediate surgical intervention may substantially decrease morbidity and mortality rates (Recommendation 1C).*

Gallbladder perforation is an unusual complication; occasionally, acute cholecystitis, inflammation, and fulminant infection may progress to ischemic necrosis and gallbladder perforation. Prompt surgical intervention is important in decreasing morbidity and mortality rates associated with this situation. The reported incidence of gallbladder perforation in acute cholecystitis is 2–11% [190–200], and mortality in such cases is as high as 12–16% [201–204].

The gallbladder perforation is classified into three types: acute or type I-free perforation with generalized peritonitis, subacute or type II-pericholecystic abscess with localized peritonitis, and chronic or type III-cholecysto-enteric fistula [205]. Perforation of the fundus is usually free perforation leading to generalized peritonitis whereas perforation in the region of body or neck becomes covered with omentum leading to localized collection. Type I and II perforations are reported to occur in a younger age group (around 50 years) whereas type III perforations are commonly seen in more elderly patients [200]. Type I perforations are typically encountered in patients with severe systemic disease (DM, atherosclerotic heart disease) without past history of acute cholecystitis, while type III perforation cases usually have previous history of recurrent attacks of cholecystitis [202–205]. The diagnosis is difficult and often delayed since the presentation is very much similar to acute cholecystitis. The ultrasound findings in such cases are also similar to the findings of acute cholecystitis, but visualization of the sonographic “hole sign” in the gallbladder wall can hint at the diagnosis of perforated gallbladder [206]. CT scan is more reliable in making the diagnosis as it better demonstrates the defect in the gallbladder wall in addition to pericholecystic collection and free intra-peritoneal fluid [206, 207].

Perforation is rarely diagnosed pre-operatively. Delayed surgical intervention is associated with elevated morbidity and mortality rates, increased likelihood of ICU admission, and prolonged post-operative hospitalization [208–210].

## Acute cholangitis

Acute cholangitis is an infectious disease characterized by acute inflammation and infection in the bile ducts resulting from a combination of biliary obstruction and bacterial growth in bile.

Bacteria reach the biliary system either by ascent from the intestine or by the portal venous system [211]. The most common cause of cholangitis is choledocholithiasis [212].

The key elements of therapy in acute cholangitis are adequate antimicrobial treatment to avoid or manage the septic complications and biliary decompression to restore biliary drainage in case of obstruction [213]. The clinical presentation varies, and initial risk stratification is important to guide further management [214].

In severe cholangitis, an early interventional approach is absolutely essential for survival.

The type and timing of biliary drainage should be based on the severity of the clinical presentation, and the availability and feasibility of drainage techniques, such as endoscopic retrograde cholangiopancreatography (ERCP), percutaneous transhepatic cholangiography (PTC), and open surgical drainage.

ERCP plays a central role in the management of biliary obstruction in patients with acute cholangitis.

*Endoscopic retrograde cholangiopancreatography (ERCP) is the treatment of choice for biliary decompression in patients with moderate/severe acute cholangitis (Recommendation 1A)* ***.***

A randomized controlled trial (RCT) [215] was conducted to compare endoscopic and open drainage in 82 patients with severe acute cholangitis with hypotension and disturbed consciousness. This RCT demonstrated that the morbidity and mortality of endoscopic naso-biliary drainage (ENBD) + endoscopic sphincterotomy (EST; *n* = 41) were significantly lower than those of T-tube drainage under laparotomy (*n* = 41). The authors concluded that morbidity and mortality of endoscopic naso-biliary drainage (ENBD) + endoscopic sphincterotomy are lower than those of T-tube drainage under laparotomy.

There are various endoscopic transpapillary options available, including biliary stent or nasobiliary drain placement above the obstruction site ± sphincterotomy, all of which with their appropriate indications corresponding to disease severity and clinical context [216].

Endoscopic biliary decompression by nasobiliary catheter or indwelling stent was equally effective for patients with acute suppurative cholangitis caused by bile duct stones in a prospective randomized trial published in 2002 [217]. The indwelling stent was associated with less post-procedure discomfort and avoided the potential problem of inadvertent removal of the nasobiliary catheter.

*Percutaneous biliary drainage (PTBD) should be reserved for patients in whom ERCP fails (Recommendation 1B).*

There are patients in whom ERCP fails because of unsuccessful biliary cannulation, or an inaccessible papilla. In these cases, percutaneous biliary drainage (PTBD) is required. However, PTBD can lead to significant complications, including biliary peritonitis, hemobilia, pneumothorax, hematoma, liver abscesses, and patient discomfort related to the catheter [218].

In 2012, a retrospective study comparing the safety and effectiveness of endoscopic and percutaneous transhepatic biliary drainage in the treatment of acute obstructive suppurative cholangitis was reported. It confirmed the clinical efficacy of endoscopic drainage as well as its ability to facilitate subsequent endoscopic or surgical intervention [219].

*Open drainage should only be used in patients for whom endoscopic or percutaneous trans-hepatic drainage is contraindicated or those in whom it has been unsuccessfully performed (Recommendation 2C)* ***.***

The indication for emergent open operation for acute cholangitis is rapidly disappearing. Emergency operation for severe cholangitis carries high mortality rates.

Given the shortened length of hospitalization and the rarity of serious complications such as intra-peritoneal hemorrhage and biliary peritonitis, endoscopic drainage is preferred to open drainage [218–220].

## Post-operative peritonitis

Post-operative peritonitis (PP) is a life-threatening hospital-acquired intra-abdominal infection with high rates of mortality [221, 222]. The most common cause of PP is an anastomotic leakage [223]. It is most frequent after rectal resection [224], but it may complicate any gastrointestinal anastomosis. Treating patients with post-operative peritonitis requires supportive therapy of organ dysfunction, source control of infection, and intensive antimicrobial therapy. The diagnosis of post-operative peritonitis may be difficult because there are no absolutely specific clinical signs and laboratory tests to reject or confirm the diagnosis. The atypical clinical presentation may be responsible for a delay in diagnosis and re-intervention or reoperation.

*On the basis of the clinical conditions, the size of the abscess and the access to interventional radiology, antibiotics and/or percutaneous drainage may be suggested to treat post-operative localized intra-abdominal abscesses when there are no signs of generalized peritonitis (Recommendation 2C).*

Antibiotics and drainage may be the optimal means of treating post-operative localized intra-abdominal abscesses when there are no signs of generalized peritonitis. Several retrospective studies in the fields of surgery and radiology have documented the effectiveness of percutaneous drainage in the treatment of post-operative localized intra-abdominal abscesses [225].

*Prompt surgical source control should be performed following diagnosis of post-operative peritonitis. Ineffective control of the septic source is associated with significantly elevated mortality rates (Recommendation 1C).*

Complete surgical source control should be performed as soon as the patient has been maximally resuscitated. The inability to control the septic source is associated with an intolerably high patient mortality [222]. Organ failure and/or subsequent re-laparotomies that have been delayed for more than 24 h both result in higher rates of mortality for patients affected by post-operative intra-abdominal infections [226]. Early re-laparotomies appear to be the most effective means of treating post-operative peritonitis [227].

In 2009, a retrospective study by Chichom-Mefire et al. [228] analyzed aspects of re-operative abdominal surgery in an economically disadvantaged environment with respect to indications, operative findings, treatment modalities, and outcomes. Mortality in this series was 18%, increasingly significant when the initial operative procedure was for peritonitis and re-operation was due to septic complications. Operative re-intervention based on clinical findings was considered the favored strategy.

## Pelvic inflammatory disease

Pelvic inflammatory disease (PID) is an infection of the upper part of the female reproductive genital tract, including the uterus, fallopian tubes, and adjacent pelvic structures and may spread to the abdomen causing peritonitis [229], caused by bacterial infection spreading from the vagina and cervix. Occasionally, right-upper quadrant pain suggestive of inflammation and adhesion formation in the liver capsule (Fitz-Hugh–Curtis syndrome) can accompany pelvic inflammatory disease.

The sexually transmitted *Neisseria gonorrhoeae* and *Chlamydia trachomatis* are present in many cases; however, microorganisms including the endogenous vaginal and cervical flora may also cause PID. Genital tract mycoplasmas, most importantly *Mycoplasma genitalium*, have recently also been implicated as a cause of acute PID [230]. The global epidemiologic profile of pelvic inflammatory disease has not been well defined. Due to financial and logistic reasons, pelvic inflammatory disease prevention programs that are based on screening are simply unavailable in most countries, where the burden of pelvic inflammatory disease may be the greatest [231].

*Patients with tubo-ovarian abscess that does not respond to antibiotics should undergo surgical drainage (Recommendation 1C).*

Tubo-ovarian abscesses (TOA) may be a complication of PID. In women of reproductive age, TOA is one of the most common types of pelvic abscess. TOA are classically treated with broad-spectrum antibiotics. However, if antibiotic therapy is not sufficient, surgical drainage should be performed [232–235].

## Post-traumatic gastrointestinal perforations

Trauma continues to be a global major public health problem worldwide, and it is associated with high morbidity and mortality worldwide regardless of the socioeconomic status [236]. Both blunt and penetrating forces may result in bowel injury; motor vehicle crashes remain the most common, and falls the next most common, cause of blunt force trauma globally [237].

Hollow visceral injury (HVI) has a more insidious presentation in this setting, often resulting in delayed diagnoses. Clinical signs may take time to develop, and imaging investigations are not completely sensitive. In addition, other injuries may distract the patient and clinical team, and accurate and timely diagnosis is often difficult. An improved outcome is reported in these settings, where, as a result of advances in imaging modalities, patient monitoring devices and prompt intervention is possible, while poor diagnostic facilities, late presentation, as well as late intervention adversely may affect the outcome in other settings [238, 239].

Several mechanisms of bowel injury have been documented in the wake of blunt abdominal trauma. The most common injury is the posterior crushing of the bowel segment between the seat belt and vertebra or pelvis. It can result in local lacerations of the bowel wall, mural and mesenteric hematomas, transection of the bowel, localized devascularization, and full-thickness contusions. Devitalization of the areas of contusion may subsequently result in late perforation. Colonic injuries occurred less frequently than small intestinal injuries perhaps due to its location and the lack of redundancy, which prevents the formation of closed loops. Abdominal trauma may be associated with other additional co-morbid injuries, which could complicate the management and affect the outcome. Delay in diagnosis and treatment of the HVI may result in early peritonitis, hemodynamic instability and increased mortality and morbidity.

*Early surgical intervention is recommended in case of HVI (Recommendation 1C).*

*Repair or anastomosis of intestinal injuries should be considered in all patients. A complete diversion of the faecal stream could be considered in colorectal injuries involving all layers in the setting of multiple injuries or comorbid conditions (Recommendation 1C).*

Early clinical recognition and surgical intervention is importance in case of HVI [239–241]. The accuracy of clinical examination signs in this setting remain poor. Signs include ecchymosis of the abdominal wall, increasing abdominal pain and distension are all associated with HVI [239]. A range of investigative modalities is available, including imaging techniques (plain x-ray, ultrasound, CT), and diagnostic peritoneal aspirate/lavage. However, in the presence of clinical peritonitis, surgical exploration is mandatory. Repair or anastomosis of intestinal injuries should be considered in all patients. A complete diversion of the fecal stream should be considered in colorectal injuries involving all layers in the setting of multiple injuries and resultant physiological compromise, unfavorable comorbid conditions, and perhaps in the setting of delayed diagnoses [239].

Damage control laparotomy (DCL) in the context of HVI is accepted for small bowel injury in the context of coagulopathy, while colon ligation has been debated because of high complication rates and an increased incidence of leakage; however, delayed anastomosis of colon injuries after DCL may avoid stoma creation in some patients who are not candidates for anastomosis during initial intervention [242].

## Re-laparotomy strategy

Severe infection may be associated with marked inflammatory responses, which in the extreme circumstance may result in an excessive, dysfunctional immune response, with resultant physiological collapse. These shocked patients develop organ dysfunction and progress to a multiple organ dysfunction syndrome (MODS). In this case, a staged operative approach may minimize further physiological insults associated with a time and energy intense primary definitive operative strategy [243].

Apart from a staged operative approach, the clinical team may also employ a planned re-laparotomy approach to re-examine pathology and facilitate repeated debridement of contaminated tissues. However, deciding if and when to perform a re-laparotomy in cases of secondary peritonitis remains difficult. Factors indicative of progressive or persistent organ failure during early post-operative follow-up analysis are the best indicators of ongoing infection [52]. Three relaparotomy strategies are currently employed for management of abdominal sepsis following an initial laparotomy: (a) open abdomen, (b) planned re-laparotomy, and (c) on-demand re-laparotomy.

*The on-demand re-laparotomy is recommended for patients with severe peritonitis because its ability to streamline healthcare resources, reduce overall medical costs, and prevent the need for further re-laparotomies (Recommendation 2A).*

In 2007, van Ruler et al. published a randomized, clinical study comparing planned and on-demand re-laparotomy strategies for patients with severe peritonitis [243]. Patients in the on-demand relaparotomy group did not have a significantly lower rate of death or major peritonitis-related morbidity compared with the planned relaparotomy group, but did have a substantial reduction in relaparotomies, healthcare utilization, and medical costs. More recently, a study conducted over a 30-month period in South Africa analyzed prospectively gathered data entered into an established electronic registry comparing patients requiring planned laparotomy (PR) with patients requiring on-demand laparotomy [244]. A total of 162 patients were included, with an average age of 36 years (standard deviation 17) and 69% male predominance. Patients selected for the PR strategy had higher admission pulse rates, higher Modified Early Warning System (MEWS) scores and significantly higher rates of diffuse intra-abdominal sepsis at initial laparotomy.

*The open abdomen may be a viable option for treating physiologically deranged patients with ongoing sepsis, facilitating subsequent exploration and control of abdominal contents, and preventing abdominal compartment syndrome (Recommendation 1C).*

*In order to define the role of OA with negative pressure therapy for improved biomediator clearance and mitigated systemic sepsis in patients with severe peritonitis a prospective trial is needed.*

The OA concept is closely linked to damage control surgery. In patients with ongoing sepsis, an OA approach may be required for controlling any persistent source of infection and preventing abdominal compartment syndrome.

OA facilitates repeated abdominal exploration in the patients with severe peritonitis allowing easy second-look to control the source of infection and evacuate inflamed and toxic content, reducing the load of peritoneal cytokines and other inflammatory substances and preventing their production by removing the source itself [245].

Temporary closure of the abdomen may be achieved by using gauze and large, impermeable, self-adhesive membrane dressings, both absorbable and non-absorbable meshes, and negative pressure therapy devices. The first and easiest method to perform a laparostomy was the application of a plastic silo (the “Bogota bag”). This system is inexpensive. However, it does not provide sufficient traction to the wound edges and allows the fascial edges to retract laterally, resulting in difficult fascial closure under significant tension, especially if the closure is delayed [243–247].

At present, negative pressure techniques (NPT) have become the most extensively employed means of temporary closure of the abdominal wall.

In 1986, Schein [246] et al. described a management technique of the open abdominal wound. It consisted of a “sandwich” composed of a Marlex mesh and an Op-Site wound dressing with interposition of suction tubes. Brock et al. in 1995 [248] described the placement of a fenestrated polyethylene sheet between the abdominal viscera and parietal peritoneum, followed by a moist towel, Kerlix gauze bandage rolls with closed suction drains or a sponge covered with an occlusive adhesive drape [245]. This method defined as the “Vacuum Pack Technique” is inexpensive, easily applied and changed, protects the viscera, prevents adhesions, removes exudate, and prevents some loss of domain [247]. Commercially prepared negative pressure dressings are available, and the initial dressing may be changed to commercial dressing, if early closure is impossible.

*Rapid closure with the assistance of negative pressure therapy should be the primary objective in the management of patients with open abdomen, in order to prevent severe morbidity such as fistulae, loss of domain and massive incisional hernias (Recommendation 1B).*

Severe complications including loss of the abdominal domain, fistula formation, and the development of giant incisional hernias may be observed in this procedure. Following re-exploration, the goal should be the early and definitive closure of the abdomen, in order to reduce the complications associated with an open abdomen. Early definitive closure (within 4–7 days of the initial laparostomy) is the basis of preventing or reducing the risk of complications [248–250].

A systematic review and meta-analysis to evaluate whether early fascial abdominal closure had advantages over delayed approach was published in 2014 [251]. The study confirmed the clinical advantages of early fascial closure compared with delayed closure in treatment of patients with open abdomen.

If unable to close the abdomen early, a progressive closure device may be necessary.

A systematic review and meta-analysis of the open abdomen and temporary abdominal closure techniques in non-trauma patients was recently published [252]. The best results in terms of achieving delayed fascial closure and reducing the risk of entero-atmospheric fistula were shown for NPT with continuous fascial traction. Despite that the authors concluded that the overall quality of the available evidence was poor, and uniform recommendations cannot be made.

## Antimicrobial therapy

Judicious use of antimicrobials is an integral part of good clinical practice. This attitude affects therapeutic efficacy of treatment and minimizes the risks associated with the selection of resistant pathogens.

Antimicrobial resistance poses a global challenge. No single country can protect itself from the importation of resistant pathogens through travel and trade.

The global nature of antimicrobial resistance calls for a global response, both in the geographic sense and across the whole range of sectors involved. Nobody is exempt from the problem [253].

Although most surgeons are aware of the problem of antimicrobial resistance, most underestimate this problem in their own hospital. The necessity of formalized systematic approaches to the optimization of antibiotic therapy for patients with intra-abdominal infections in the setting of surgical units worldwide has become increasingly urgent.

*Knowledge of regional/local rates of resistance, when it is available, should be always an essential component of the clinical decision-making process when deciding the empirical treatment of infection (Recommendation 1C).*

Regional epidemiological data and resistance profiles are essential for selecting appropriate antibiotic therapy for IAIs [254, 255].

However, while high-income countries (HICs) have extensive surveillance systems to monitor antimicrobial resistance [255], in low- and middle-income countries LMIC surveillance systems have not really been established.

The Study for Monitoring Antimicrobial Resistance Trends (SMART) provides the best available evidence for the current status of cIAIs worldwide. The SMART has monitored the in vitro susceptibility patterns of clinical gram-negative bacilli to antimicrobial agents collected worldwide from intra-abdominal infections since 2002 [256, 257]. Isolates worldwide showed the highest levels of antimicrobial resistance of the global regions included the SMART study, and a trend of increasing resistance continues year by year. One particular cause for concern is the prevalence of extended-spectrum β-lactamase (ESBL)-producing Enterobacteriaceae in the clinical setting. The prevalence of ESBLs intra-abdominal infections has steadily increased over time for in Asia. Europe, Latin America, Middle East, North America, and South Pacific [256, 257].

In addition to the expected increased resistance to beta-lactams, fluoroquinolone resistance in ESBL-positive *Escherichia coli* causing intra-abdominal infections ranges from 60 to 93% in India, China, North America, Europe, and South Africa [256, 257]. Although carbapenem activity against isolates from IAIs is also high, it is slightly lower than activity against *Klebsiella pneumoniae* isolates from urinary tract infections.

*Predicting the pathogens and potential resistance patterns of a given infection begins by establishing whether the infection is community-acquired or healthcare-associated.*

F*or patients with community-acquired intra-abdominal infections (CA-IAIs), agents with a narrower spectrum of activity are preferred. However, in CA-IAI patients at risk for extended-spectrum beta-lactamases (ESBLs) producing Enterobacteriaceae infections, anti-ESBL-producer coverage may be warranted. For patients with healthcare-associated infections (HA-IAIs), antibiotic regimens with broader spectra of activity are preferred (Recommendation 1B).*

Initial antibiotic therapy for IAIs is typically empirical in nature because a patient with abdominal sepsis needs immediate treatment, and microbiological data (culture and susceptibility results) can require up to 48–72 h before they are available for a more detailed analysis. Selection of appropriate empiric antibiotic therapy is critical for preventing unnecessary morbidity and mortality from cIAIs. The major pathogens involved in community-acquired intra-abdominal infections are usual residents of gastrointestinal flora, including *Enterobacteriaceae*, streptococci, and certain anaerobes (particularly *Bacteroides fragilis*) [75]. Narrower spectrum antimicrobial agents are appropriate for these patients [258].

In the context of intra-abdominal infections, the main resistance problem is posed by ESBL-producing *Enterobacteriaceae*, which are prevalent in hospital-acquired infections but observed in CA-IAIs too [139].

Specific risk factors for ESBL-producing bacteria in community-acquired infections include recent exposure to antibiotics (particularly third generation cephalosporins or fluoroquinolones) within 90 days of IAI or known colonization with ESBL producing *Enterobacteriaceae* [253].

Although increasing overtime everywhere, carriage of ESBL producing *Enterobacteriaceae* did not evolve with the same dynamics and large intra- and inter-regional variations have been observed. Poor access to drinking water, water pollution, and a high population density are efficient drivers for ESBL producing *Enterobacteriaceae* dissemination, as for any fecally-orally transmitted diseases [259]. Reports from the Western Pacific, Eastern Mediterranean, and Southeast Asia regions showed the highest carriage rates and the most striking recent ascending trends [259], explaining why travelers into these areas of the world are at risk of becoming colonized [260]. In contrast, rates reported in Europe never exceeded 10% [259].

HA IAIs include hospital-acquired infections (developing greater than 48 h after initial source control) but also infections in patients having recent hospitalization within 90 days, living in a skilled nursing or other long-term care facility, using aggressive medical therapies (intravenous therapy, wound dressing) at home and invasive therapies (haemodialysis, chemotherapy, radiotherapy) in outpatient clinics within 30 days of the index infection.  HA-IAIs are commonly caused by more resistant flora. Here, complex multidrug regimens may be necessary for first line, empiric therapy. Although transmission of multidrug resistant organisms is most frequently documented in acute care facilities, all healthcare settings are affected by the emergence and transmission of antimicrobial-resistant microbes [253].

Resistant flora may include the non-fermenting gram-negative *Pseudomonas aeruginosa*, very dangerous either in the abdominal cavity, [261] or in hepatobiliary surgery [262–265] and *Acinetobacter spp*, ESBL-producing *K. pneumonia*, *E. coli* and *vancomycin*-resistant enterococci (VRE) [266, 267].

During the last two decades, antimicrobial resistance has become a global threat to public health systems and some of the most common causes include erroneous use of antibiotics, and poor prevention and control with respect to infections. Particularly, infections caused by resistant gram-negative bacteria [253, 268] are becoming increasingly prevalent and now constitute a serious threat to public health worldwide because they are difficult to treat and are associated with high morbidity and mortality rates. Bacteria producing carbapenemases, such as *K. pneumonia*, are rapidly emerging as a major source of multidrug-resistant infections worldwide [269–272] and pose a serious threat in clinical situations where administration of effective empiric antibiotics is essential to prevent mortality following bacteraemia and infections in immunocompromised patients. Non-fermenting gram-negative bacteria (*P. aeruginosa*, *Stenotrophomonas maltophilia*, and *Acinetobacter baumannii*) have exhibited alarming rates of increased resistance to a variety of antibiotics in health facilities worldwide. Both species are intrinsically resistant to several drugs and could acquire additional resistance to other important antimicrobial agents [253]. *P. aeruginosa* coverage is only generally recommended for patients with HA-IAIs.

Among gram-positive bacteria, enterococci play a significant role in IAI. Some studies have demonstrated poor outcomes among patients with documented enterococcal infections, particularly in those with post-operative IAI where enterococci coverage should be always considered [273, 274].

The acquisition of glycopeptide resistance by enterococci has seriously affected the treatment and control of these organisms. Affected patients usually have multiple and relevant co-morbidities, with prolonged hospital stay and received long courses of broad-spectrum antibiotics [274].

The burden of multidrug-resistant organism (MDRO) infections in LMICs is difficult to quantify because in these countries, routine microbiologic culture and sensitivity testing, especially in rural hospitals, are not performed because of the lack of personnel, equipment, and financial resources. As a consequence, antimicrobial therapy is empirical and a small collection of antimicrobials may be overused. This approach, although relatively inexpensive, may increase the emergence of antimicrobial resistance and hence sub-optimal clinical outcomes [253].

Therefore, although resistance containment interventions in healthcare structures have mostly been implemented in high-income countries, there is a pressing need to intervene in the resistance pandemic also in LMIC.

In the setting of HA-IAIs, post-operative peritonitis (PP) is a life-threatening hospital-acquired intra-abdominal infection with high rates of mortality and high risk for MDR infections and invasive candidiasis [275, 276]. Antimicrobial therapy between initial intervention and reoperation seems to be a significant risk factor for emergence of MDRO in patients with PP. A study by Augustin et al. [276] included all consecutive adult patients with a diagnosis of post-operative peritonitis requiring admission to a surgical intensive care unit from January 2001 to December 2004. A total of 269 bacteria were cultured in 100 patients including 41 episodes with MDRO. According to logistic regression analysis, the use of broad-spectrum antibiotics between the initial intervention and reoperation was a significant risk factor for emergence of MDRO.

In 2015, a retrospective review of patients with anastomotic leakage after colorectal cancer surgery [277], reported the use of antibiotics for more than 5 days before diagnosis of anastomosis site leakage, and diabetes mellitus were identified as independent risk factors for MDRO acquisition by multivariate analysis.

*In critically ill patients antimicrobial therapy should be started as soon as possible.*

*In these patients to ensure timely and effective administration of antibiotics, clinicians should always consider the pathophysiological status of the patient as well as the pharmacokinetic properties of the employed antibiotics (Recommendation 1B).*

An ineffective or otherwise inadequate antimicrobial regimen is one of the variables more strongly associated with unfavorable outcomes in critical ill patients [278].

Empiric antimicrobial therapy should be started as soon as possible in patients with organ dysfunction and septic shock [279].

However, apart from early timing of administration, selection of a pharmacological agent with penetration to the site of presumed infection, is necessary. Furthermore, the pathophysiological and immunological status of the patient and the pharmacokinetic properties of the chosen drugs warrant consideration. In the event of abdominal sepsis, clinicians must be aware that drug pharmacokinetics may be altered significantly in critically ill patients due to the pathophysiology of sepsis. For example, in critically ill patients, higher than standard loading doses of hydrophilic antimicrobials such as beta-lactams should be administered to ensure optimal exposure at the infection site independently of the patient’s renal function because of the dilution effect [280].

Two patterns of bactericidal activity have been described for antibiotics: time-dependent activity (where the time that the plasma concentration persists above the MIC of the etiological agent is considered the major determinant for efficacy) and concentration-dependent activity (where the efficacy is mainly related to the plasma peak concentration in relation to the MIC of the microorganism). The efficacy of time-dependent antibacterial agents in severely ill patients is related primarily to the maintenance of supra-inhibitory concentrations, and therefore, multiple daily dosing or continuous infusion may be appropriate [280, 281].

On the other hand, some agents including aminoglycosides have concentration-dependent activity; therefore, for this antibiotic class, the entire daily dose should be administered in a once daily way (or with the lowest possible number of daily administrations) to achieve the highest peak plasma level and reduce the renal cortex exposure to aminoglycosides and reduces the risk of nephrotoxicity [282].

*In patients with uncomplicated IAI such as uncomplicated appendicitis and uncomplicated cholecystitis, where the source of infection is treated definitively, post-operative antibiotic therapy is not necessary* (*Recommendation 1A).*

*In patients with complicated IAI undergoing an adequate source-control procedure, a short course of antibiotic therapy (3-5 d) is always recommended (Recommendation 1A).*

*Patients who have ongoing signs of peritonitis or systemic illness (ongoing infection) beyond 5 to 7 days of antibiotic treatment, should warrant a diagnostic investigation (Recommendation 1C).*

Antibiotics should be used after a treatable infection has been recognized or if there is a high degree of suspicion of an infection. In the setting of uncomplicated acute IAIs such as uncomplicated appendicitis or cholecystitis, single doses have the same impact as multiple doses and post operative antimicrobial therapy is not necessary if source control is adequate [170, 283–285].

In the setting of cIAIs, a short course of antibiotic therapy (3–5 days) after adequate source control is a reasonable option [170, 283–285]. The recent prospective trial by Sawyer et al. demonstrated that in patients with cIAI undergoing an adequate source control, the outcomes after approximately 4 days fixed-duration antibiotic therapy were similar to those after a longer course of antibiotics that extended until after the resolution of physiological abnormalities [286]. However, in critically ill patients with ongoing sepsis, an individualized approach should be always mandatory and patient’s inflammatory response should be monitored regularly [287] and decisions to continue, narrow, or stop antimicrobial therapy must be made on the basis of clinician judgment.

Patients who have ongoing signs of peritonitis or systemic illness beyond 5–7 days of antibiotic treatment normally warrant a diagnostic investigation to determine whether additional surgical intervention is necessary to address an ongoing uncontrolled source of infection or antimicrobial treatment failure. The prolonged and inappropriate use of antibiotics appears a key factor in the rapid rise of antimicrobial resistance worldwide over the past decade [287]. A rational and appropriate use of antibiotics is particularly important both to optimize quality clinical care and to reduce selection pressure on resistant pathogens. Several strategies aiming at achieving optimal use of antimicrobial agents have been described, but it is important that surgeons know antibiotic administration minimal requirements. Without these minimal requirements, surgeons worldwide will increase the likelihood of treatment failures and antibiotic resistance.

*The choice of empiric antibiotic regimens in patients with IAI should be based on the clinical condition of the patients, the individual risk for infection by resistant pathogens, and the local resistance epidemiology (Recommendation 1C).*

Intra-abdominal infections may be managed with either single or multiple antibiotic regimens.

Beta-lactam/beta-lactamase inhibitor combinations have an in vitro activity against gram-positive, gram-negative, and anaerobe organisms. Amoxicillin/clavulanate is still an option in mild community acquired IAIs. Broad-spectrum activity of piperacillin/tazobactam, including anti-*P. seudomonas* effect and anaerobic coverage, still make it an interesting option for management of severe IAIs. However, the use of piperacillin/tazobactam in patients with ESBLs infections is still controversial [288, 289], even if in stable patients, it may be still a therapeutic chance.

Third generation cephalosporins including cefotaxime and ceftriaxone in association with metronidazole, may be still options for the treatment of mild IAIs. Ceftazidime and cefoperazone are third generation cephalosporins with an activity against *P. aeruginosa*. Cefepime, is a fourth-generation cephalosporin, with broader spectrum activity than third generation cephalosprins and effective against AmpC-producing organisms [290]. For empiric therapy, also cefepime should also be combined with metronidazole because it does not possess anti-anaerobic activity [291].

Ciprofloxacin and levofloxacin are no longer appropriate choice as first-line treatment in many geographic regions because of the prevalence of fluoroquinolone resistance. However, when employed, these drugs should be used in association with metronidazole. In many current practices, the fluoroquinolones remain available for patients presenting allergy to beta-lactams, with mild intra-abdominal infections [75].

Carbapenems offer a wide spectrum of antimicrobial activity against gram-positive and gram-negative aerobic and anaerobic pathogens (with the exception of MDR-resistant gram-positive cocci). Group 1 carbapenems include ertapenem. This group has activity against extended-spectrum beta-lactamase (ESBL)-producing pathogens, but not active against *P. aeruginosa* and *Enterococcus* species. Group 2 includes imipenem/cilastatin, meropenem, and doripenem, which share activity against non-fermentative gram-negative bacilli [75].

For more than two decades, carbapenems have been considered the agents of choice for multidrug-resistant infections caused by *Enterobacteriaceae*. The recent and rapid spread *K. pneumoniae* carbapenems resistant has become a critical issue in hospitals worldwide. The use of carbapenems should be limited so as to preserve activity of this class of antibiotics because of the concern of emerging carbapenem-resistance [253].

Other options include aminoglycosides, particularly for suspected infections by gram-negative bacteria. They are effective against *P. aeruginosa*, but are ineffective against anaerobic bacteria and need association with metronidazole. Because of their toxic side effects, some guidelines did not recommend aminoglycosides for the routine empiric treatment of community-acquired IAI, reserving them for patients with allergies to beta-lactam agents or in combination with beta-lactams for treatment of IAI with suspected MDR gram-negative bacteria [292].

Tigecycline is a viable treatment option, especially in empiric therapy, for complicated IAIs due to its favorable in vitro activity against anaerobic organisms, enterococci, several ESBL- and in association carbapenemase-producing *Enterobacteriaceae*, *Acinetobacter* species, and *Stenotrophomonas maltophilia* [293–295]. It does not feature in vitro activity against *P. aeruginosa* or *P. mirabilis*. Caution is always advised for its use, in suspected bacteremia and healthcare-associated pneumonia [296].

The recent challenges of treating multidrug-resistant gram-negative infections, especially in critically ill patients, have renewed interest in the use of “old” antibiotics such as polymyxins and fosfomycin [297, 298], now routinely used for treatment of MDR bacteria in critical ill patients.

Ceftolozone/tazobactam and ceftazidime/avibactam are new antibiotics that have been approved for treatment of cIAIs (in combination with metronidazole) including infection by ESBLs producing *Enterobacteriaceae* and *P. aeruginosa*. These antimicrobials will be valuable for treating infections caused by MDR gram-negative bacteria in order to preserve carbapenems [299]. Ceftolozone/tazobactam has excellent in vitro activity against MDR *P. aeruginosa* [299]. Ceftazidime/avibactam seems to have an in vitro activity against *K. pneumoniae* carbapenemase-producing bacteria [300]. Although many reviews have been written, their precise role as empiric treatment for complicated IAI remains to be defined [301].

In table 4, antibiotics for treating patients with cIAIs as proposed by the AGORA working group are illustrated [253]. Appendices 1, 2, 3, 4 list antimicrobial regimens for management of patients with cIAIs in different settings worldwide.

**Table 4:** Antibiotics for treating patients with IAIs based upon susceptibility [253]

Antibiotic	Enterococci	Ampicillin-resistant enterococci	Vancomycin-resistantenterococci	*Enterobacteriaceae*	ESBL-producing *Enterobactericeae*	*Pseudomonas aeruginosa*	Anaerobic gram-negative bacilli
Penicillins/beta-lactamase inhibitors							
Amoxicillin/clavulanate	+	−	−	+	−	−	+
Ampicillin/sulbactam	+	−	−	+	−	−	+/−
Piperacillin/tazobactam	+	−	−	+	+/−	+	+
Carbapenems							
Ertapenem	−	−	−	+	+	−	+
Imipenem/cilastatin	+/−^a^	−	−	+	+	+	+
Meropenem	−	−	−	+	+	+	+
Doripenem	−	−	−	+	+	+	+
Fluoroquinolones							
Ciprofloxacin	−	−	−	+	−	+^b^	−
−−Levofloxacin	+/−	−	−	+	−	+/−	−
Moxifloxacin	+/−	−	−	+	−	−	+/−
Cephalosporins							
Ceftriaxone	−	−	−	+	−	−	−
Ceftazidime	−	−	−	+	−	+	−
Cefepime	−	−	−	+	+/−	+	−
Ceftozolane/tazobactam	−	−	−	+	+	+	−
Ceftazidime/avibactam	−	−	−	+	+	+	−
Aminoglycosides							
Amikacin				+	+	+	−
Gentamicin				+	+	+	−
Glycylcyclines							
Tigecycline	+	+	+	+^c^	+	−	+
5-Nitroimidazole							
Metronidazole							+
Polymixyn							
Colistimethate (Colistin)	−	−	−	+^d^	+	+	−
Glycopeptides							
Teicoplanin	+	+	−	−	−	−	−
Vancomycin	+	+	−	−	−	−	−
Oxazolidines							
Linezolid	+	+	+	−	−	−	−

^a^Imipenem/cilastatin is more active against ampicillin-susceptible enterococci than ertapenem, meropen and doripenem

^b^Ciprofloxacin is more active against *P. aeruginosa* than levofloxacin

^c^Not active against *Proteus*, *Morganella*, and *Providencia*

^d^Not active against *Morganella*, *Proteus*, *Providencia*, *Salmonella*, *Serratia*, *Shigella*, and *Yersina* (*Y. enterocolitica*)

*Intra-operative cultures should be always performed in patients with HA-IAs or with CA- at risk for resistant pathogens or in critically ill patients. They allow expansion of the antimicrobial regimen if the initial choice is too narrow and to perform a de-escalation if the empirical regimen is too broad.*

*When a microorganism is identified in clinical cultures, antimicrobial susceptibility testing (AST) should always be performed and reported to guide antibiotic therapy (Recommendation 1C).*

Although a lack of impact on patient outcomes by bacteriological cultures has been documented in patients with community-acquired IAI, especially in appendicitis [302, 303], the results of microbiological testing may have great importance for the choice of therapeutic strategy of every patient, in particular, in the adaptation of targeted antimicrobial treatment in patients at risk of unpredictable organisms.

Obtaining microbiological results from intra-operative culture from the site of infection has two advantages: (a) to expand antimicrobial regimen if the initial choice was too narrow and (b) to perform de-escalation of antimicrobial therapy if the empirical regimen was too broad [253].

When a microorganism is identified in clinical cultures, antimicrobial susceptibility testing (AST) should always be performed to guide antimicrobial therapy. Data are reported in the form of MIC, which is the lowest concentration of an antibiotic that inhibits visible growth of a microorganism.

The numerical MIC number, expressed as micrograms/ml, is usually reported by microbiology laboratories as a categorical guide for clinicians, i.e., as “susceptible”, “resistant”, or “intermediate,” according to Clinical or Laboratory Standards Institute (CLSI) criteria in the USA or the European Committee for Antimicrobial Susceptibility Testing (EUCAST) criteria in Europe.

*Knowledge of mechanisms of secretion of antibiotics into bile may be helpful in designing the optimal therapeutic regimen for patients with biliary-related intra-abdominal infections (Recommendation 1C).*

Organisms most often isolated in biliary infections are those isolated in intra-abdominal infections including the gram-negative aerobes, *E. coli* and *K. pneumonia* and anaerobes, especially *B. fragilis* [153]. The role of enterococci in biliary tract infections remains unclear, and specific coverage against these microorganisms is not routinely suggested for community-acquired biliary infections [153].

Although there are no clinical data to support the use of antibiotics with biliary penetration for these patients, the efficacy of antibiotics in the treatment of biliary infections may depend on effective biliary antibiotic concentrations too [153]. Obviously in patients with obstructed bile ducts, the biliary penetration of antibiotics may be poor and effective biliary concentrations are reached only in a minority of patients [153].

Antibiotics commonly used to treat biliary tract infections and their biliary penetration ability are illustrated in Table 5.

**Table 5:** Antibiotics commonly used to treat biliary tract infections and their biliary penetration ability [153]

Good penetration efficiency	Low penetration efficiency
Piperacillin/tazobactam	Ceftriaxone
Tigecycline	Cefotaxime
Amoxicillin/clavulanate	Meropenem
Ciprofloxacin	Ceftazidime
Ampicillin/sulbactam	Vancomycin
Cefepime	Amikacin
Levofloxacin	Gentamicin
Imipenem	

*Empirical antifungal therapy for Candida species is recommended for patients with hospital-acquired IAIs, especially those with recent abdominal surgery or anastomotic leak (Recommendation 1C).*

The epidemiological profile of *Candida spp* in the context of nosocomial peritonitis is incompletely defined. Its clinical presence is usually associated with poor prognosis. Empirical antifungal therapy for *Candida spp* is typically not recommended for patients with community-acquired intra-abdominal infections, with the notable exceptions of critically ill patients or immunocompromised patients (due to neutropenia or concurrent administration of immunosuppressive agents, such as glucocorticosteroids, chemotherapeutic agents, and immunomodulators) [304]. Recently, IDSA guidelines for the treatment of invasive candidiasis were developed and addressed *Candida* peritonitis [275]. IDSA guidelines suggested considering empiric antifungal therapy for patients with clinical evidence of intra-abdominal infection and significant risk factors for candidiasis, including recent abdominal surgery, anastomotic leaks, or necrotizing pancreatitis, who are doing poorly despite treatment for bacterial infections.

Preferred empiric therapy in critically ill patients or those previously exposed to an azole is an echinocandin (Caspofungin: loading dose of 70 mg, then 50 mg daily; Micafungin:100 mg daily; Anidulafungin: loading dose of 200 mg, then 100 mg daily). However, fluconazole, 600 mg (12 mg/kg) loading dose, then 400 mg (6 mg/kg) daily, should be still considered first-line antifungal therapy, in hemodynamically stable patients who are colonized with azole susceptible *Candida* species or who have no prior exposure to azoles [275].

## Conclusions

IAIs remain an important cause of morbidity and mortality in modern surgical practice worldwide. The cornerstones of effective treatment of IAIs include early and accurate diagnosis, prompt resuscitation, early and effective source control, and initiation of appropriate antimicrobial therapy. IAIs have different disease and clinical spectra in the various regions of the world, and their etiological factors show a wide geographical variation. Promoting standards of care in the managing IAIs worldwide should be mandatory to grant management guidelines to all surgeons.

In Appendix 5, all the WSES recommendations are illustrated.

## Appendix 1

### Empiric antibiotic regimens for non-critically ill patients with community-acquired IAIs. Normal renal function

*Community-acquired cIAIs*

*Non-critically ill patients*

**Amoxicillin/clavulanate 1.2-2.2 g 6-hourly**

or

**Ceftriazone 2 g 24-hourly + Metronidazole 500 mg 6-hourly**

or

**Cefotaxime 2g 8-hourly + Metronidazole 500 mg 6-hourly**

or

In patients with beta-lactam allergy

**Ciprofloxacin 400 mg 8-hourly + Metronidazole 500 mg 6- hourly**

or

**Moxifloxacin 400 24-hourly**

or

In patients at risk for infection with community-acquired ESBL-producing Enterobacteriacea

**Ertapenem 1 g 24 hourly**

or

**Tigecycline 100 mg initial dose, then 50 mg 12-hourly**

## Appendix 2

### Empiric antibiotic regimens for critically il l patients with community-acquired IAIs. Normal renal function

*Community-acquired IAIs*

*Critically ill patients*

**Piperacillin/Tazobactam 4.5 g 6-hourly**

or

**Cefepime 2 g 8-hourly + Metronidazole 500 mg 6-hourly**

or

In patients at risk for infection with community-acquired ESBL-producing Enterobacteriacea

**Meropenem 1 g 8-hourly**

or

**Doripenem 500 mg 8-hourly**

or

**Imipenem/Cilastatin 1 g 8-hourly**

In patients at high risk for infection with Enterococci including immunocompromised patients or patients with recent antibiotic exposure consider use of **Ampicillin 2 g 6-hourly** if the patients are not being treated with piperacillintazobactam or imipenem-cilastatin (active against ampicillin-susceptible enterococci)

## Appendix 3

### Empiric antimicrobial regimens for non-critically ill patients with healthcare-associated IAIs. Normal renal function (CrCl>90 mL/min)

*Healthcare-associated IAIs*

*Non-critically ill patients*

**Piperacillin/Tazobactam 4.5 g 6-hourly**

or

In patients at higher risk for infection with MDROs including recent antibiotic exposure, patient living in a nursing home or long-stay care with an indwelling catheter, or post-operative IAI

**Meropenem 1 g 8-hourly + Ampicillin 2 g 6-hourly**

or

**Doripenem 500 mg 8-hourly + Ampicillin 2 g 6-hourly**

or

**Imipenem/Cilastatin 1 g 8-hourly**

or

As a carbapenem-sparing regimen

**Piperacillin/Tazobactam 4.5 g 6-hourly + Tigecycline 100 mg initial dose, then 50 mg 12-hourly**

+/-

In patients at high risk for invasive candidiasis

**Fluconazole 800 mg LD then 400 mg 24-hourly**

In patients with documented beta-lactam allergy consider use of antibiotic combinations with **Amikacin 15–20 mg/kg 24-hourly**

## Appendix 4

### Empiric antimicrobial regimens for critically ill patients with healthcare-associated IAIs. Normal renal function

*Healthcare-associated IAIs*

*Critically ill patients*

**Meropenem 1 g 8-hourly**

or

**Doripenem 500 mg 8-hourly**

or

**Imipenem/Cilastatin 1 g 8-hourly**

or

As a carbapenem-sparing regimen

**Ceftolozane /Tazobactam 1.5 g 8-hourly + Metronidazole 500 mg 6-hourly**

or

**Ceftazidime/Avibactam 2.5 g 8-hourly + Metronidazole 500 mg 6-hourly**

+

**Vancomycin 25–30 mg/kg loading dose then 15–20 mg/kg/dose 8-hourly**

or

**Teicoplanin 12 mg/kg 12-hourly times 3 loading dose then 12 mg/kg 24-hourly**

or

In patients at risk for infection with vancomycin-resistant enterococci (VRE) including patients with previous enterococcal infection or colonization, immunocompromised patients, patients with long ICU stay, or recent Vancomycin exposure

**Linezolid 600 mg 12-hourly**

or

**Daptomycin 6 mg/kg 24-hourly**

+-

In patients at high risk for invasive candidiasis

**Echinocandins: caspofungin (70 mg LD, then 50 mg daily), anidulafungin (200 mg LD, then 100 mg daily), micafungin (100 mg daily) or Amphotericin B Liposomal 3 mg/kg/dose 24-hourly**

In patients with suspected or proven infection with MDR (non-metallo-beta-lactamase-producing) Pseudomonas aeruginosa consider use of antibiotic combinations with **Ceftolozane /Tazobactam**

In patients with suspected or proven infection with carbapenemase-producing Klebsiella pneumoniae consider use of antibiotic combinations with **Ceftazidime/Avibactam**

In patients with documented beta-lactam allergy consider use of antibiotic combinations with **Amikacin 15–20 mg/kg 24-hourly**

## Appendix 5

### WSES recommendations for management of intra-abdominal infections

**Principles of sepsis control**

#### Statement 1

Early recognition of the patient with ongoing abdominal sepsis is an essential step for an effective treatment.

Prompt administration of intravenous fluids for resuscitation is critical in patients with an ongoing sepsis. This initial resuscitation should be titrated to the clinical response, and not solely guided by a predetermined protocol. Vasopressor agents may serve to augment and assist fluid resuscitation, particularly where this therapy alone is failing (Recommendation 1A).

### Diagnosis

#### Statement 2

A step-up approach for diagnosis from clinical and laboratory examination, to imaging examination should be used and tailored to the hospitals resources (Recommendation 1B).

### Source control

#### Statement 3

Appendectomy remains the treatment of choice also for acute appendicitis. Antibiotic therapy is a safe means of primary treatment for patients with uncomplicated acute appendicitis, but it is less effective in the long-term due to significant recurrence rates and probably needs the certainty of a CT proven diagnosis of uncomplicated appendicitis (Recommendation 1A).

#### Statement 4

Both open and laparoscopic appendectomies are viable approaches to surgical treatment of acute appendicitis (Recommendation 1A).

#### Statement 5

Patients with a periappendiceal abscess can be managed with percutaneous image-guided drainage in surgical departments with ready access to diagnostic and interventional radiology. When percutaneous drainage is not available, surgery is suggested (Recommendation 1B).

#### Statement 6

In patients treated conservatively, interval appendectomy may be not necessary following initial non-operative treatment of complicated appendicitis. However, interval appendectomies should always be performed for patients with recurrent symptoms (Recommendation 2B).

#### Statement 7

Routine use of intra-operative irrigation for appendectomies does not prevent intra-abdominal abscess formation and may be avoided (Recommendation 2B).

#### Statement 8

Antibiotics can be avoided in patients with CT findings of uncomplicated ALCD and without significant comorbid conditions or signs of sepsis. Patients should be clinically monitored to assess for resolution of the inflammatory processes (Recommendation 1A).

#### Statement 9

On the basis of clinical conditions, patients with diverticular smaller abscesses may be treated by antibiotics alone (Recommendation 1C).

#### Statement 10

Patients with abscesses having a large diameter should be treated by percutaneous drainage and intravenous antibiotics (Recommendation 1C).

#### Statement 11

Whenever percutaneous drainage of the abscess is not feasible or not available, based on the clinical conditions patients with large abscesses can be initially treated by antibiotic therapy alone. However, careful clinical monitoring is mandatory (Recommendation 1C).

#### Statement 12

Hartmann’s procedure remains useful in the management of diffuse peritonitis in critically ill patients. However, in clinically stable patients, primary resection with anastomosis, with or without a diverting stoma, may be performed (Recommendation 1B).

#### Statement 13

Laparoscopic peritoneal lavage and drainage may not be considered the treatment of choice in patients with diffuse peritonitis (Recommendation 1A).

#### Statement 14

Treatments for perforated colonic carcinoma should both stabilize the emergency condition of the peritonitis and fulfil the technical objectives of oncological intervention (Recommendation 1B).

#### Statement 15

Patients presenting with diffuse peritonitis caused by colonoscopic perforation should undergo immediate surgical intervention, which typically involves primary repair or resection (Recommendation 1B).

#### Statement 16

An early laparoscopic approach may be a safe and effective option for colonoscopy-related colonic perforation for experienced surgeons (Recommendation 2B).

#### Statement 17

Surgery is the treatment of choice for perforated peptic ulcers (Recommendation 1A).

#### Statement 18

Simple closure with or without an omental patch is a safe and effective procedure to address small perforated ulcers (<2 cm) (Recommendation 1A).

#### Statement 19

Laparoscopic repair of perforated peptic ulcers can be a safe and effective procedure for experienced surgeons (Recommendation 1A).

#### Statement 20

Surgery is the treatment of choice for patients with small bowel perforations (Recommendation 1B).

#### Statement 21

In the event of small perforations, primary repair is recommended (Recommendation 1B).

#### Statement 22

In the case of abdominal tuberculosis perforation, resection of the affected area and anastomosis may be the treatment of choice rather than primary closure (Recommendation 1C).

#### Statement 23

Early cholecystectomy is a safe treatment for acute cholecystitis and generally results in shorter recovery time and hospitalization compared to delayed cholecystectomies (Recommendation 1A).

#### Statement 24

Laparoscopic cholecystectomy is a safe and effective treatment for acute cholecystitis (Recommendation 1A).

It is the first choice for patients with acute cholecystitis where adequate resources and skill are available. Some risk factors may predict the risk for conversion to open cholecystectomy.

#### Statement 25

Cholecystostomy is a safe and effective treatment for acute cholecystitis in critically ill and/or with multiple comorbidities and unfit for surgery patients (Recommendation 1B).

#### Statement 26

Early diagnosis of gallbladder perforation and immediate surgical intervention may substantially decrease morbidity and mortality rates (Recommendation 1C).

#### Statement 27

Endoscopic retrograde cholangiopancreatography (ERCP) is the treatment of choice for biliary decompression in patients with moderate/severe acute cholangitis (Recommendation 1A)**.**

#### Statement 28

Percutaneous biliary drainage (PTBD) should be reserved for patients in whom ERCP fails (Recommendation 1B).

#### Statement 29

Open drainage should only be used in patients for whom endoscopic or percutaneous trans-hepatic drainage is contraindicated or those in whom it has been unsuccessfully performed (Recommendation 2C).

#### Statement 30

On the basis of the clinical conditions, the size of the abscess and the access to interventional radiology, antibiotics, and/or percutaneous drainage may be suggested to treat post-operative localized intra-abdominal abscesses when there are no signs of generalized peritonitis (Recommendation 2C).

#### Statement 31

Prompt surgical source control should be performed following diagnosis of post-operative peritonitis. Ineffective control of the septic source is associated with significantly elevated mortality rates (Recommendation 1C).

#### Statement 32

Patients with tubo-ovarian abscess that does not respond to antibiotics should undergo surgical drainage (Recommendation 1C).

#### Statement 33

Early surgical intervention is recommended in case of HVI (Recommendation 1C).

#### Statement 34

Repair or anastomosis of intestinal injuries should be considered in all patients. A complete diversion of the fecal stream could be considered in colorectal injuries involving all layers in the setting of multiple injuries or comorbid conditions (Recommendation 1C).

#### Statement 35

The on-demand re-laparotomy is recommended for patients with severe peritonitis because its ability to streamline healthcare resources reduce overall medical costs and prevent the need for further re-laparotomies (Recommendation 2A).

#### Statement 36

The open abdomen may be a viable option for treating physiologically deranged patients with ongoing sepsis, facilitating subsequent exploration and control of abdominal contents, and preventing abdominal compartment syndrome (Recommendation 1C).

In order to define the role of OA with negative pressure therapy for improved biomediator clearance and mitigated systemic sepsis in patients with severe peritonitis, a prospective trial is needed.

#### Statement 37

Rapid closure with the assistance of negative pressure therapy should be the primary objective in the management of patients with open abdomen, in order to prevent severe morbidity such as fistulae, loss of domain, and massive incisional hernias (Recommendation 1B).

### Antimicrobial therapy

#### Statement 38

Knowledge of regional/local rates of resistance, when it is available, should be always an essential component of the clinical decision-making process when deciding the empirical treatment of infection (Recommendation 1C).

#### Statement 39

Predicting the pathogens and potential resistance patterns of a given infection begins by establishing whether the infection is community-acquired or hospital-acquired.

For patients with community-acquired intra-abdominal infections (CA-IAIs), agents with a narrower spectrum of activity are preferred. However, in CA-IAI patients at risk for extended-spectrum beta-lactamases **(**ESBLs) producing *Enterobacteriaceae* infections, anti-ESBL-producer coverage may be warranted. For patients with hospital-acquired infections (HA-IAIs), antibiotic regimens with broader spectra of activity are preferred (Recommendation 1B).

#### Statement 40

In critically ill patients, antimicrobial therapy should be started as soon as possible.

In these patients, to ensure timely and effective administration of antibiotics, clinicians should always consider the pathophysiological status of the patient as well as the pharmacokinetic properties of the employed antibiotics (Recommendation 1B).

#### Statement 41

In patients with uncomplicated IAI such as uncomplicated appendicitis and uncomplicated cholecystitis, where the source of infection is treated definitively, post-operative antibiotic therapy is not necessary (Recommendation 1A).

#### Statement 42

In patients with complicated IAI undergoing an adequate source-control procedure, a short course of antibiotic therapy (3–5 days) is always recommended (Recommendation 1A).

#### Statement 43

Patients who have ongoing signs of peritonitis or systemic illness (ongoing infection) beyond 5–7 days of antibiotic treatment should warrant a diagnostic investigation (Recommendation 1C).

#### Statement 44

The choice of empiric antibiotic regimens in patients with IAI should be based on the clinical condition of the patients, the individual risk for infection by resistant pathogens, and the local resistance epidemiology (Recommendation 1C).

#### Statement 45

Intra-operative cultures should be always performed in patients with HA-IAs or with CA- at risk for resistant pathogens or in critically ill patients. They allow expansion of the antimicrobial regimen if the initial choice is too narrow and to perform a de-escalation if the empirical regimen is too broad.

When a microorganism is identified in clinical cultures, antimicrobial susceptibility testing (AST) should always be performed and reported to guide antibiotic therapy (Recommendation 1C).

#### Statement 46

Knowledge of mechanisms of secretion of antibiotics into bile may be helpful in designing the optimal therapeutic regimen for patients with biliary-related intra-abdominal infections (Recommendation 1C).

#### Statement 47

Empirical antifungal therapy for *Candida* species is recommended for patients with hospital-acquired IAIs, especially those with recent abdominal surgery or anastomotic leak (Recommendation 1C).

## Abbreviations

CA-IAIs: Community-acquired intra-abdominal infections
HA-IAIs: Hospital-acquired intra-abdominal infections
IAIs: Intra-abdominal infections
MDRO: Multidrug-resistant organism
