# Erratum to: The management of intra-abdominal infections from a global perspective: 2017 WSES guidelines for management of intra-abdominal infections

**DOI:** 10.1186/s13017-017-0148-z

**Published:** 2017-08-02

**Authors:** M. Sartelli, A. Chichom-Mefire, F. M. Labricciosa, T. Hardcastle, F. M. Abu-Zidan, A. K. Adesunkanmi, L. Ansaloni, M. Bala, Z. J. Balogh, M. A. Beltrán, O. Ben-Ishay, W. L. Biffl, A. Birindelli, M. A. Cainzos, G. Catalini, M. Ceresoli, A. Che Jusoh, O. Chiara, F. Coccolini, R. Coimbra, F. Cortese, Z. Demetrashvili, S. Di Saverio, J.J. Diaz, V. N. Egiev, P. Ferrada, G. P. Fraga, W. M. Ghnnam, J. G. Lee, C. A. Gomes, A. Hecker, T. Herzog, J. I. Kim, K. Inaba, A. Isik, A. Karamarkovic, J. Kashuk, V. Khokha, A. W. Kirkpatrick, Y. Kluger, K. Koike, V. Y. Kong, A. Leppaniemi, G. M. Machain, R. V. Maier, S. Marwah, M. E. McFarlane, G. Montori, E. E. Moore, I. Negoi, I. Olaoye, A. H. Omari, C. A. Ordonez, B. M. Pereira, G. A. Pereira Júnior, G. Pupelis, T. Reis, B. Sakakushev, N. Sato, H. A. Segovia Lohse, V. G. Shelat, K. Søreide, W. Uhl, J. Ulrych, H. Van Goor, G.C. Velmahos, K. C. Yuan, I. Wani, D. G. Weber, S. K. Zachariah, F. Catena

**Affiliations:** 1 Department of Surgery, Macerata Hospital, Macerata, Italy; 2 Department of Surgery and Obstetrics/Gynaecology, Regional Hospital, Limbe, Cameroon; 3 Department of Biomedical Sciences and Public Health, Unit of Hygiene, Preventive Medicine and Public Health, Università Politecnica delle Marche, Ancona, Italy; 4 Trauma Service, Inkosi Albert Luthuli Central Hospital and Department of Surgery, Nelson R Mandela School of Clinical Medicine, Durban, South Africa; 5 Department of Surgery, College of Medicine and Health Sciences, UAE University, Al-Ain, United Arab Emirates; 6 Department of Surgery, College of Health Sciences, Obafemi Awolowo University, Ile-Ife, Nigeria; 7 General Surgery Department, Papa Giovanni XXIII Hospital, Bergamo, Italy; 8 Trauma and Acute Care Surgery Unit, Hadassah Hebrew University Medical Center, Jerusalem, Israel; 9 Department of Traumatology, John Hunter Hospital and University of Newcastle, Newcastle, NSW Australia; 10 Department of General Surgery, Hospital San Juan de Dios de La Serena, La Serena, Chile; 11 Department of General Surgery, Rambam Health Care Campus, Haifa, Israel; 12 Acute Care Surgery at The Queen’s Medical Center, John A. Burns School of Medicine, University of Hawai’i, Honolulu, USA; 13 Department of Surgery, Maggiore Hospital, Bologna, Italy; 14 Department of Surgery, Hospital Clínico Universitario, Santiago de Compostela, Spain; 15 Department of General Surgery, Kuala Krai Hospital, Kuala Krai, Kelantan Malaysia; 16 Emergency Department, Niguarda Ca’ Granda Hospital, Milan, Italy; 17 Department of Surgery, UC San Diego Medical Center, San Diego, USA; 18 Emergency Surgery Unit, San Filippo Neri’s Hospital, Rome, Italy; 19 Department of Surgery, Tbilisi State Medical University, Kipshidze Central University Hospital, T’bilisi, Georgia; 20 Shock Trauma Center, University of Maryland School of Medicine, Baltimore, USA; 21 Department of Surgery, Pirogov Russian National Research Medical University, Moscow, Russian Federation; 22 Department of Surgery, Virginia Commonwealth University, Richmond, VA USA; 23 Division of Trauma Surgery, Department of Surgery, School of Medical Sciences, University of Campinas (Unicamp), Campinas, SP Brazil; 24 Department of General Surgery, Mansoura Faculty of Medicine, Mansoura University, Mansoura, Egypt; 25 Department of Surgery, Yonsei University College of Medicine, Seoul, Republic of Korea; 26 Department of Surgery, Hospital Universitário Terezinha de Jesus, Faculdade de Ciências Médicas e da Saúde de Juiz de Fora, Juiz de Fora, Brazil; 27 Department of General and Thoracic Surgery, University Hospital Giessen, Giessen, Germany; 28 Department of Surgery, St. Josef Hospital, Ruhr University Bochum, Bochum, Germany; 29 Department of Surgery, Ilsan Paik Hospital, Inje University College of Medicine, Goyang, Republic of Korea; 30 Division of Acute Care Surgery and Surgical Critical Care, Department of Surgery, Los Angeles County and University of Southern California Medical Center, University of Southern California, Los Angeles, CA USA; 31 Department of General Surgery, Faculty of Medicine, Erzincan University, Erzincan, Turkey; 32 Clinic for Emergency Surgery, Medical Faculty University of Belgrade, Belgrade, Serbia; 33 Department of Surgery, Assia Medical Group, Tel Aviv University Sackler School of Medicine, Tel Aviv, Israel; 34 Department of Emergency Surgery, Mozyr City Hospital, Mozyr, Belarus; 35 Departments of Surgery, Critical Care Medicine, and the Regional Trauma Service, Foothills Medical Centre, Calgary, AB Canada; 36 Department of General Surgery, Division of Surgery, Rambam Health Care Campus, Haifa, Israel; 37 Department of Primary Care and Emergency Medicine, Kyoto University Graduate School of Medicine, Kyoto, Japan; 38 Department of Surgery, Edendale Hospital, Pietermaritzburg, South Africa; 39 Abdominal Center, University Hospital Meilahti, Helsinki, Finland; 40 II Cátedra de Clínica Quirúrgica, Hospital de Clínicas, Facultad de Ciencias Medicas, Universidad Nacional de Asuncion, Asuncion, Paraguay; 41 Department of Surgery, University of Washington, Seattle, WA USA; 42 Department of Surgery, Pt BDS Post Graduate Institute of Medical Sciences, Rohtak, India; 43 Department of Surgery, Radiology, University Hospital of the West Indies, Kingston, Jamaica; 44 Department of Surgery, University of Colorado, Denver Health Medical Center, Denver, CO USA; 45 Department of Surgery, Emergency Hospital of Bucharest, Bucharest, Romania; 46 Department of Surgery, University of Ilorin, Teaching Hospital, Ilorin, Nigeria; 47 Department of Surgery, King Abdullah University Hospital, Irbid, Jordan; 48 Department of Surgery and Critical Care, Universidad del Valle, Fundación Valle del Lili, Cali, Colombia; 49 Division of Emergency and Trauma Surgery, Ribeirão Preto Medical School, Ribeirão Preto, Brazil; 50 Department of General and Emergency Surgery, Riga East University Hospital ‘Gailezers’, Riga, Latvia; 51 Emergency Post-operative Department, Otavio de Freitas Hospital and Hosvaldo Cruz Hospital, Recife, Brazil; 52 General Surgery Department, Medical University, University Hospital St George, Plovdiv, Bulgaria; 53 Department of Aeromedical Services for Emergency and Trauma Care, Ehime University Graduate School of Medicine, Ehime, Japan; 54 Department of General Surgery, Tan Tock Seng Hospital, Tan Tock Seng, Singapore; 55 Department of Gastrointestinal Surgery, Stavanger University Hospital, Stravenger, Norway; 56 Department of Clinical Medicine, University of Bergen, Bergen, Norway; 57 First Department of Surgery - Department of Abdominal, Thoracic Surgery and Traumatology, General University Hospital, Prague, Czech Republic; 58 Department of Surgery, Radboud University Medical Center, Nijmegen, The Netherlands; 59 Trauma, Emergency Surgery, and Surgical Critical Care Harvard Medical School, Massachusetts General Hospital, Boston, USA; 60 Trauma and Emergency Surgery Department, Chang Gung Memorial Hospital, Taoyuan City, Taiwan; 61 Department of Surgery, Sheri-Kashmir Institute of Medical Sciences, Srinagar, India; 62 Department of Trauma Surgery, Royal Perth Hospital, Perth, Australia; 63 Department of Surgery, Mosc Medical College, Kolenchery, Cochin India; 64 Department of Emergency Surgery, Maggiore Hospital, Parma, Italy

## Erratum

The original article [1] contained an error whereby a co-author, Boris Sakakushev had their name spelt incorrectly.

The original article has now been updated to display Dr. Sakakushev’s name correctly.
